# Integrative transcriptome-proteome approach reveals key hypoxia-related features involved in the neuroprotective effects of Yang Xue oral liquid on Alzheimer’s and Parkinson’s disease

**DOI:** 10.3389/fphar.2024.1411273

**Published:** 2024-07-09

**Authors:** Xiang-Yang Chen, Ming-Rong Cheng, Chen-Chen Tang, Chen-Qin Xu, Yi-Lang Zhong, Yuan Gao, Xue-Xiang Cheng, Jian Chen

**Affiliations:** ^1^ College of Life and Environment Science, Huangshan University, Huangshan, Anhui, China; ^2^ Nanxiang Branch of Ruijin Hospital, Shanghai, China; ^3^ Department of Experimental Management, School of Integrative Medicine, Shanghai University of Traditional Chinese Medicine, Shanghai, China; ^4^ Department of Vascular Disease, Shanghai TCM-Integrated Hospital, Shanghai University of Traditional Chinese Medicine, Shanghai, China; ^5^ Traditional Chinese Recovery and Treatment Center, Zhejiang Rehabilitation Medical Center, Hangzhou, China; ^6^ College of Pharmacy, Hubei University of Chinese Medicine, Wuhan, China; ^7^ Department of Public Health, International College, Krirk University, Bangkok, Thailand

**Keywords:** Alzheimer’s disease, Parkinson’s disease, transcriptome, proteome, signature

## Abstract

**Introduction:** This study investigates the role of hypoxia-related genes in the neuroprotective efficacy of Yang Xue oral liquid (YXKFY) in Alzheimer’s disease (AD) and Parkinson’s disease (PD).

**Methods and results:** Using differential expression and weighted gene co-expression network analysis (WGCNA), we identified 106 and 9 hypoxia-associated genes in AD and PD, respectively, that are implicated in the transcriptomic and proteomic profiles. An artificial intelligence-driven hypoxia signature (AIDHS), comprising 17 and 3 genes for AD and PD, was developed and validated across nine independent cohorts (*n* = 1713), integrating 10 machine learning algorithms and 113 algorithmic combinations. Significant associations were observed between AIDHS markers and immune cells in AD and PD, including naive CD4^+^ T cells, macrophages, and neutrophils. Interactions with miRNAs (hsa-miR-1, hsa-miR-124) and transcription factors (USF1) were also identified. Single-cell RNA sequencing (scRNA-seq) data highlighted distinct expression patterns of AIDHS genes in various cell types, such as high expression of TGM2 in endothelial cells, PDGFRB in endothelial and mesenchymal cells, and SYK in microglia. YXKFY treatment was shown to repair cellular damage and decrease reactive oxygen species (ROS) levels. Notably, genes with previously dysfunctional expression, including FKBPL, TGM2, PPIL1, BLVRB, and PDGFRB, exhibited significant recovery after YXKFY treatment, associated with riboflavin and lysicamine.

**Conclusion:** The above genes are suggested to be central to hypoxia and neuroinflammation responses in AD and PD, and are potential key mediators of YXKFY’s neuroprotective action.

## Introduction

Alzheimer’s disease (AD) and Parkinson’s disease (PD) are the two most common neurodegenerative disorders that have a significant impact on the aging population worldwide (Nowell et al., 2023). AD is estimated to affect approximately 4% of individuals aged 65 and above, whereas PD is estimated to affect around 1% of the total population of individuals aged 60 and above (Macdonald et al., 2018; Aborode et al., 2022). AD is characterized by progressive memory loss, cognitive decline, and the accumulation of amyloid-beta (Aß) plaques and neurofibrillary tangles in the brain (Breijyeh and Karaman, 2020). PD, on the other hand, is characterized by motor symptoms such as tremors, rigidity, and bradykinesia, as well as the loss of dopaminergic neurons in the substantia nigra (Hayes, 2019). While the etiology of AD and PD remains complex and multifactorial, emerging evidence suggests that hypoxia is detrimental to the brain and plays a crucial role in the pathogeneses of these diseases (Burtscher et al., 2021). Hypoxia can result from various factors, including impaired blood flow, mitochondrial dysfunction, and oxidative stress, all of which have been implicated in the development and progression of AD and PD (Grabska-Kobyłecka et al., 2023; Su et al., 2023). Understanding the role of hypoxia-related features in the pathogenesis of AD and PD is crucial for the development of novel therapeutic strategies. Targeting hypoxia signaling pathways, such as HIF-1α, may offer potential therapeutic avenues for the treatment and prevention of these devastating neurodegenerative disorders (Baillieul et al., 2017).

Traditional Chinese medicines (TCMs) have been used in the treatment of neurological disease for thousands of years (Su et al., 2024). Yangxue oral liquid (YXKFY) is composed of four traditional Chinese medicines: Melanteritum, Fructus Crataegi, Fructus Hippophae, and Ziziphus jujuba. Since 7000 B.C., the fruit of Crataegus pinnatifida has been used to produce prehistoric fermented beverages (Moreira et al., 2023). Recent studies have shown that the fruit possesses neuroprotective properties that could be effective against AD (Lee et al., 2019; Moreira et al., 2023). Fructus Hippophae has been traditionally used in the treatment of brain conditions, with the aim of enhancing cognitive abilities such as learning and memory, as well as alleviating pathological damage in mice with AD through modulating oxidative stress and inflammatory processes (Dinkar Gore et al., 2023; Zhao et al., 2023). The fruit of Ziziphus jujuba has been proven to possess anti-inflammatory (Al-Reza et al., 2010) and neuroprotective (Kaeidi et al., 2015) abilities due to the presence of neuroprotective compounds such as triterpenoids, flavonoids, polysaccharides, saponins and alkaloids (Kim et al., 2021). Hence, YXKFY exhibits a promising potential for therapeutic intervention in the treatment of neurodegenerative diseases. Nevertheless, further research is needed to clarify the material basis and targets of YXKFY in addressing neurodegenerative diseases.

Recently, there has been a growing body of evidence supporting the widespread use of transcriptomics and proteomics in enhancing our understanding of pathophysiological mechanisms and facilitating the development of diagnostic tools for various diseases (Li et al., 2020; Mu et al., 2020; Yang et al., 2021; Yang et al., 2023). Moreover, the utilization of weighted gene coexpression network analysis (WGCNA) offers the ability to illustrate the interconnections between diverse genes through the creation of a co-expression network, which not only enables the identification of modules that are associated with specific phenotypes but also proves to be more efficient in the investigation of crucial pathways and genes involved in numerous human disorders (Langfelder and Horvath, 2008; Zhang et al., 2024). In addition, the limitations of expression-based multigene signatures in clinical settings can be attributed to their lack of uniqueness and appropriateness in selected modeling methods, and the absence of strict validation in large multicenter cohorts (Wang et al., 2022; Yang et al., 2024). Hence, our objective was to conduct a comprehensive analysis of the molecular mechanisms, as well as the diagnostic and therapeutic targets of YXKFY on AD/PD, using transcriptome, proteome, and hypoxia-related features.

**FIGURE 1:**
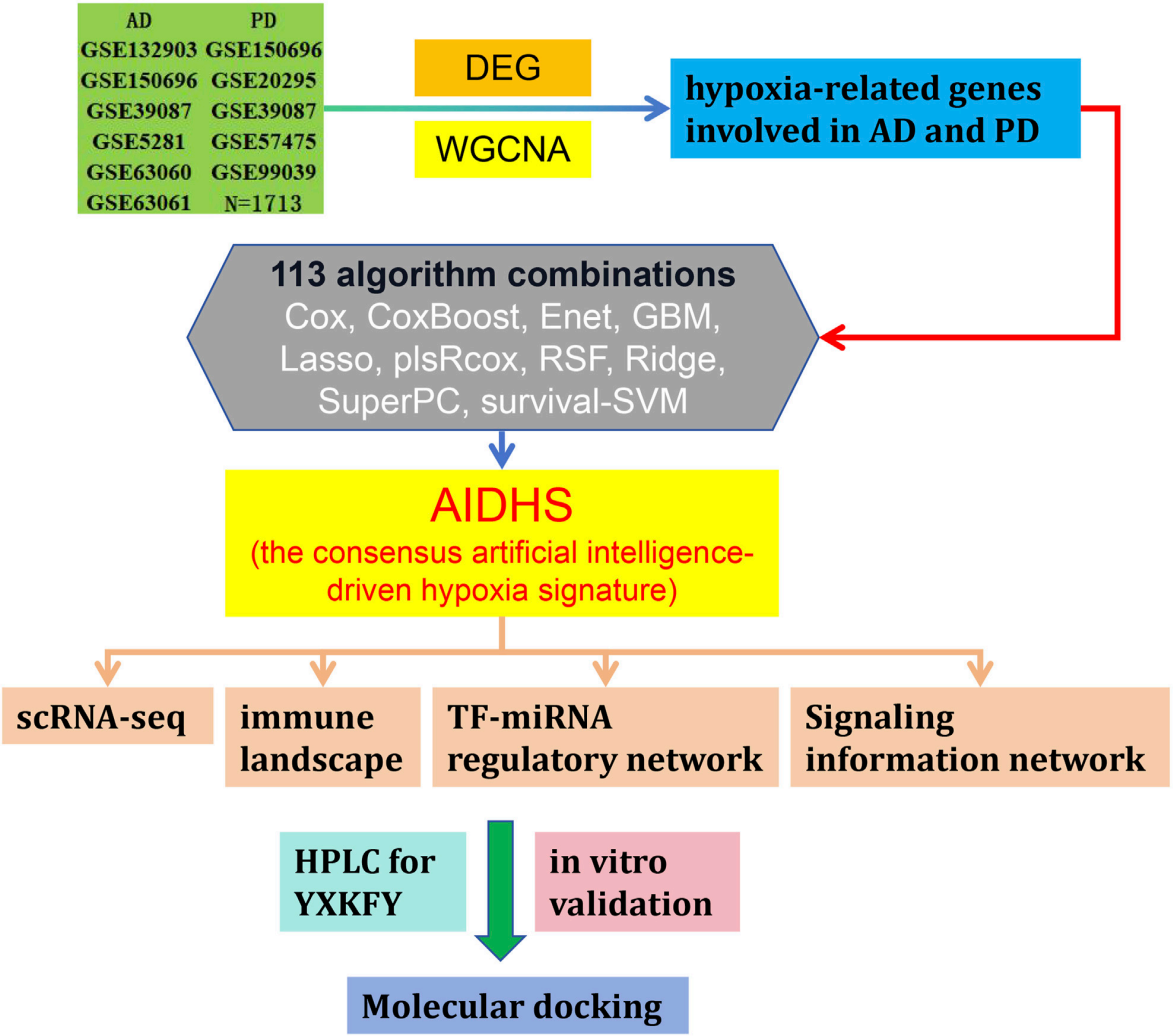
Flowchart of the study design.

In this study, we performed an integrated analysis to discovery a consensus artificial intelligence-driven hypoxia signature (AIDHS) from 110 kinds of algorithm combinations across transcriptome and proteome. Then, we employed ultra-high-performance liquid chromatography coupled with Quadrupole Exactive-Orbitrap high-resolution mass spectrometry (UHPLC-Q Exactive-Orbitrap HR-MS) to identify the chemicals present in YXKFY. Finally, the key AIDHS was applied in *in vitro* experimental validation to assess potential hypoxia-related mechanisms of YXKFY in AD/PD. Figure 1 showed the flowchart of the study design.

## Materials and methods

### Data acquisition from the GEO database

The mRNA and protein expression data for AD/PD were acquired from the Gene Expression Omnibus (GEO) database (https://www.ncbi.nlm.nih.gov/gds). A total of 36 AD, 48 PD and 47 normal serum samples were collected from GSE339087 (protein) for differential expression analysis. GSE5281 (87 AD cases and 74 controls) and GSE26927 (12 PD cases and 8 controls) datasets were selected for weighted gene co-expression network analysis. Then, we downloaded GSE150696 (9 AD, 12 PD cases and 9 controls), GSE132903 (97 AD cases and 98 controls), GSE63060 (145 AD cases and 104 controls), GSE63061 (140 AD cases and 134 controls), GSE20295 (40 PD cases and 53 controls), GSE99039 (205 PD cases and 233 controls) and GSE57475 (93 PD cases and 49 controls) for further analysis and validation.

### Data preprocessing and differential expression analysis

Various intergroup comparisons were conducted using Sangerbox 3.0 (http://vip.sangerbox.com/home.html), which is a freely accessible online platform for data analysis and visualization. Initially, normalization of protein expression in the GSE339087 dataset (AD and PD) was carried out. Following this, volcano plots and heatmaps were generated for the differentially expressed proteins (DEPs). DEPs with an absolute fold change of ≥ 1.2 and a *p*-value < 0.05 were deemed to be statistically significant in terms of differential expression.

### Weighted gene co-expression network analysis (WGCNA)

To identify modules of highly correlated genes and hub genes, we utilized the WGCNA (Langfelder and Horvath, 2008) approach to construct scale-free co-expression networks for two independent datasets: GSE5281 (87 AD cases and 74 controls) and GSE26927 (12 PD cases and 8 controls). In the GSE5281 dataset, the brain regions examined were the entorhinal cortex (EC), hippocampus (HIP), medial temporal gyrus (MTG), posterior cingulate (PC), superior frontal gyrus (SFG), and primary visual cortex (PVC). For the GSE26927 dataset, the substantia nigra (SN) was the focused brain region.

We initially employed Pearson correlation-based hierarchical clustering to group all the genes and samples. Subsequently, we determined the soft threshold power value to establish the co-expression network, ensuring that it conformed to a scale-free co-expression network. The adjacency matrices were then transformed into a topological overlap matrix to identify gene modules. Moreover, similar modules were merged and clustered together. Finally, we generated module-trait relationship diagrams and obtained the gene list associated with each module.

### Intersection of hypoxia-related genes involved in AD and PD shared by transcriptome and proteome

The top four modules that have the closest association with AD/PD were identified by analyzing the module-trait relationship diagrams of the GSE5281 and GSE26927 datasets. A total of 8,371 hypoxia-related genes (HRG) were obtained from COREMINE (https://www.coremine.com/medical/#search). To identify the common HRGs between the DEGs and the aforementioned module genes, Venn diagram analysis was conducted. The overlapping genes were then subjected to functional enrichment analysis using the Metascape (https://metascape.org/gp/index.html) (Zhou Y. et al., 2019).

### Signature obtained from artificial intelligence-driven integrative approaches

In order to ensure a highly accurate and stable performance for the consensus artificial intelligence-driven hypoxia signature (AIDHS), we integrated a total of 10 machine learning algorithms and explored 113 algorithm combinations. These integrative algorithms include Cox, CoxBoost, elastic network (Enet), generalised boosted regression modelling (GBM), Lasso, stepwise partial least squares regression for Cox (plsRcox), random survival forest (RSF), Ridge, supervised principal components (SuperPC), and survival support vector machine (survival-SVM).

The procedure for generating the signature was as follows: (a) We performed the 113 algorithm combinations on the HRGs, fitting prediction models based on the leave-one-out cross-validation (LOOCV) framework using the GSE39087 cohort; (b) We then validated all these models using nine additional datasets (GSE5281, GSE26927, GSE150696, GSE132903, GSE63060, GSE63061, GSE20295, GSE99039, and GSE57475); (d) For each model, we calculated the area under the curve (AUC) score across all validation datasets. In the case of PD, we considered the model with the highest average AUC as optimal. For AD, the optimal model was determined based on higher average AUC and the least number of genes involved.

### Profile of the immune cell infiltration

In order to assess the role of the immune microenvironment in the development of AD and PD, we analyzed and compared the immune infiltration patterns in both AD/PD and normal groups through CIBERSORTx tool (https://cibersortx.stanford.edu/), which specifically designed for accurately quantifying the relative proportions of 22 immune cells subtypes within a complex mixture of gene expressions. Lastly, to assess the correlation between hub genes and differential infiltrated immune cells, a Pearson correlation test analysis was carried out. For further investigation, a correlation threshold of |r| > 0.2 was established.

### Signaling information network and TF-miRNA regulatory networks analysis of the hub genes

SIGNOR 2.0 (https://signor.uniroma2.it/) (Licata et al., 2020), a public repository that stores manually-annotated causal relationships between proteins and other biologically relevant entities, was performed to explore the signal transduction relationship of the former hub genes. Then, TF-miRNA coregulatory network analysis was implemented on the hub genes through curated regulatory interaction information collected from the RegNetwork repository (Liu et al., 2015) with Networkanalyst online tools (Zhou G. et al., 2019).

### Analysis the heterogeneity of striata in the AD and PD single-cell transcriptome

The basal ganglia are frequently affected in AD and PD. However, there is limited understanding of the fundamental molecular processes involved. The striatum is crucial for motor learning and various cognitive functions. Therefore, the single-cell dataset GSE161045 was obtained from the GEO database (https://www.ncbi.nlm.nih.gov/), which contains 4 AD, 4 PD and 4 control striata samples. The data underwent thorough preprocessing, including the removal of cells with fewer than 200 genes, more than 5,000 genes, or more than 25% mitochondrial genes. After this process, a total of 31,030 filtered cells remained for analysis. To mitigate potential batch effects due to sample identity, we utilized the “Harmony” R package (version 0.1.0) for batch correction (Korsunsky et al., 2019). Subsequently, we used the FindNeighbors and FindClusters functions (with a resolution of 0.1) for cell cluster identification, following the uniform manifold approximation and projection (UMAP) analysis. Prior to this, we referenced preexisting markers from published literature to identify specific cell types and their respective markers through SingleCellBase (Meng et al., 2023). Finally, we present bubble plots depicting the expression of 26 AIDHS genes in different disease and cell groups.

### Serum preparation containing YXKFY

The YXKFY samples used in this study were acquired from Hubei Fenghuang Baiyunshan Pharmaceutical Co., Ltd. Blood samples containing YXKFY were obtained from the YXKFY rat group. The original concentration of the YXKFY was 0.4 g/mL. The rats were administered a dosage of 2 mg/mL of the YXKFY. Each rat received 5 mL of the YXKFY orally, once in the morning and once in the afternoon, for a total of two administrations per day. This oral administration was continued for 5 consecutive days. Following the last administration, the abdominal aorta was punctured to collect blood 1 h later. The collected blood samples were centrifuged at 3000 r/min to separate the serum, which was then incubated at 56°C for 30 min for inactivation. The serum was subsequently filtered and sterilized using a 0.22 micron filter, and stored at −20°C for future use. All of the animal procedures, such as housing and care, and experimental protocols were approved by the Ethics Committee of Shanghai University of Traditional Chinese Medicine (No. PZSHUTCM210702015).

### Cell culture and treatment

Human neuroblastoma SH-SY5Y cells were cultured in a medium composed of DMEM supplemented with 10% fetal bovine serum (FBS, Gibco, Australia) at a controlled environment of 37°C with 5% CO2. The cells were evenly distributed with a density of 10,000 cells per well in 96-well plates and allowed to grow for a period of 12 h. To determine the optimal concentration of the hypoxic model in SH-SY5Y cells, the viability of SH-SY5Y cells was assessed using the CCK8 assay after exposure to different concentrations of CoCl2 (0 μM, 25 μM, 50 μM, 100 μM, 200 μM, 400 μM, 500 μM, 600 μM, 800 μM, and 1000 μM) for 24 h. In order to evaluate the potential neuroprotective properties of YXKFY on SH-SY5Y cells, CoCl2 group was exposed to 800 μM for 24 h; and YXKFY group was subjected to 800 μM CoCl2 and serum containing 10% YXKFY for 24 h.

### ROS measurement

ROS generation in SH-SY5Y cells was examined using the fluorescent dye 2′,7′-dichlorodihydrofluorescein diacetate (DCFH-DA; Beyotime, Shanghai, China). SH-SY5Y cells were firstly washed three times with phosphate-buffered saline (PBS). Subsequently, the cells were incubated in PBS supplemented with 10 μM of DCFH-DA at a temperature of 37°C for a duration of 20 min. Following the incubation period, cells were once again washed three times using PBS. To visualize the cells, the measurements and analysis were conducted using a FACS Calibur flow cytometer (Beckman, San Jose, United States).

### Validation of YXKFY-associated AIDHS genes by RT-qPCR

The extraction of total RNA from cells was carried out using TRIzol (Invitrogen Corporation, CA, United States). Subsequently, cDNA was synthesized using the cDNA Synthesis SuperMix. In order to detect the mRNA expression levels of AIDHS, RT-qPCR was conducted using GAPDH as an internal reference. The amplification primers used in the experiment were obtained from Shanghai Sangon Biological Engineering Technology and are detailed in Table 1. To ensure consistency, the mRNA expression level of each target gene was normalized to that of GAPDH within the same sample. The relative expression of each target gene was determined using the 2^−ΔΔCT^ method.

**TABLE 1:** Primer sequence of RT-qPCR.

Gene	Forward	Reverse
BLVRB	CCT​GAA​GTA​CGT​GGC​TGT​GAT	TCA​TGT​TTG​GAG​ATG​ACC​CTT​GA
C10orf54	TCA​TCC​TGC​TCC​TGG​TCT​ACA​A	AAT​CCC​TTG​AAT​GTT​GCT​GTC​CAT
CDC37	TGA​GGT​GTC​TGA​TGA​TGA​AGA​CG	GTT​CCT​CCT​TCT​CCT​TCT​GGA​AC
CHCHD6	TCT​TCA​GAG​CAA​TTC​CAT​GAG​GC	GAT​CTC​GGT​AGC​AGT​GGA​GAA​TC
FKBPL	TTG​GAG​AAA​AGG​ACA​CCT​CTC​AG	CTT​ACT​TCC​AGC​TCA​AGC​GTT​TC
FN1	AGC​CGA​GGT​TTT​AAC​TGC​GA	CCC​ACT​CGG​TAA​GTG​TTC​CC
GLUL	GTG​AGA​AAG​TCC​AGG​CCA​TGT​AT	CTG​TTG​GAA​CCC​TCA​GAC​TGT​AA
HOXC4	GTA​TAG​CTG​CAC​CAG​TCT​CCA​G	AGA​GCG​ACT​GTG​ATT​TCT​CGG
PFN1	GCT​ACA​AGG​ACT​CGC​CCT​C	CAA​GTG​TCA​GCC​CAT​TCA​CGT​A
PPIL1	TGC​TCC​AAA​GAC​CTG​TAA​GAA​CT	TTG​CCA​TAG​ATA​GAT​GCA​CCA​CC
PRDX4	GAG​ACA​CTA​CGT​TTG​GTT​CAA​GC	TTT​CAC​TAC​CAG​GTT​TCC​AGC​C
PSMB1	CTG​CAA​TGC​TGT​CTA​CAA​TCC​TG	TCT​CTC​TGG​TAA​GAC​CCT​ACT​GG
PSMG1	GCT​AGA​AAA​ATA​TCC​GTG​CTC​CA	GTT​TAG​CAC​AAC​CAA​CTT​CCT​CC
SLC16A14	CAT​CTG​TGC​TAA​TGG​CAT​CTC​TG	CGA​ATG​CAC​GGC​TGA​ATA​AGT​AA
SLC17A7	CAT​GGT​CAA​TAA​CAG​CAC​GAC​C	ACA​ATG​TAG​CCC​CAG​AAA​AAG​GA
SMYD3	GGG​GTT​CAA​GTG​ATG​AAA​GTT​GG	TCT​TCA​ATC​AGG​CTG​TGT​TCT​CT
SYK	GCA​GAA​GCC​ATA​TCG​AGG​GA	ATC​TCT​CTT​GGA​CAC​CCT​GC
FMO5	TAG​CCA​AAC​AGC​CAA​GCA​GG	AGT​CCC​CTA​CAC​GAT​TCA​GGA
HDAC4	TCA​CTC​CCT​ACC​TGA​GCA​CC	GGC​CTG​AAA​GAT​ACC​AGT​CTG​T
TGM2	GTC​AGC​TAC​AAT​GGG​ATC​TTG​GG	AAG​GCA​GTC​ACG​GTA​TTT​CTC​AT
DNM2	TCG​ACA​TTG​AGC​AGT​CCT​ACA​TC	GGG​ATG​GCT​CTC​TTC​TTG​TTC​AG
PDGFRB	GGA​CAT​ACC​CCC​GCA​AAG​AA	CTC​TCC​GTC​ACA​TTG​CAG​GT
FXYD5	CCT​GTG​TCT​TCT​CAC​CAT​CGT​T	AGA​ACT​GGA​CGT​GGT​ATC​TTT​CA
SETDB1	GAT​GCT​GTC​AAC​AAG​AAG​AGC​AG	GCC​TTT​GTG​CCA​AGT​CTT​AGT​TC
MYBPH	TCT​CAG​AAA​ACC​TGT​GTG​GAC​TC	AGA​AGT​CTC​GCT​CAA​TAA​ACC​CT
JUP	CAA​CAA​GAA​CAA​CCC​CAA​GTT​CC	GGT​CCA​GAG​CAG​CTT​TTC​ATA​AC

### Analysis of YXKFY and drug serum via high performance liquid chromatography (HPLC)

To perform the analysis, an Acquity UPLC^®^ BEH C18 column (100 mm × 2.1 mm, 1.7 µm) was utilized in conjunction with a Dionex Ultimate 3,000 high-pressure liquid system. The mobile phase consisted of a mixture of 0.1% formic acid in water and methanol. The elution procedure involved a stepwise gradient: from 0 to 4 min, 4% methanol; from 4 to 10 min, 4%–12% methanol; from 10 to 30 min, 12%–70% methanol; at 30 min, 70% methanol; from 35 to 38 min, 70%–95% methanol; at 38 min, 95% methanol; and from 42 to 45 min, 4% methanol. The flow velocity was maintained at 0.3 mL/min. The column temperature was set at 40°C, and the sample size used for each analysis was 5 μL. For the analysis and detection of the compounds, a Q Exactive quadrupole-electrostatic field orbitrap high-resolution mass spectrometer was employed. The ion source used was an electrospray ion source (H-ESI). The scanning mode utilized for this analysis was full MS/SIM mode (covered m/z 80–1,200), allowing for the detection of both positive and negative ions.

### Molecular docking validation

The Protein Data Bank (PDB, https://www.rcsb.org/) was accessed to retrieve the crystal structures of the primary targets. The three-dimensional configurations of the active compounds were obtained from PubChem (https://pubchem.ncbi.nlm.nih.gov/). Utilizing AutoDock Vina (http://vina.scripps.edu/), molecular docking was carried out to compute the affinity between the active compounds and primary targets. The corresponding PDB identifiers were 5oog, 2 × 7 k and 3s3j for BLVRB, PPIL1, and TGM2, respectively. The AlphaFold algorithm was used to predict the structures of FKBPL and PDGFRB, which are proteins without a known 3D structure in the PDB database. Then, water molecules were eliminated from the proteins, and polar hydrogen atoms were added. Subsequently, active binding sites were constructed based on the ligand’s position in the PDB complex.

### Statistical analysis

The data presented in this study were expressed as the mean ± standard deviation (SD), using the social science statistical software package for data analysis. An independent sample t-test was employed to compare the two groups of data, while a one-way analysis of variance was used to compare multiple sets of data. The column graphs were created using GraphPad Prism 6 software. A significance level of *p* < 0.05 was considered statistically significant.

## Results

### Identification of DEPs in AD and PD

**FIGURE 2:**
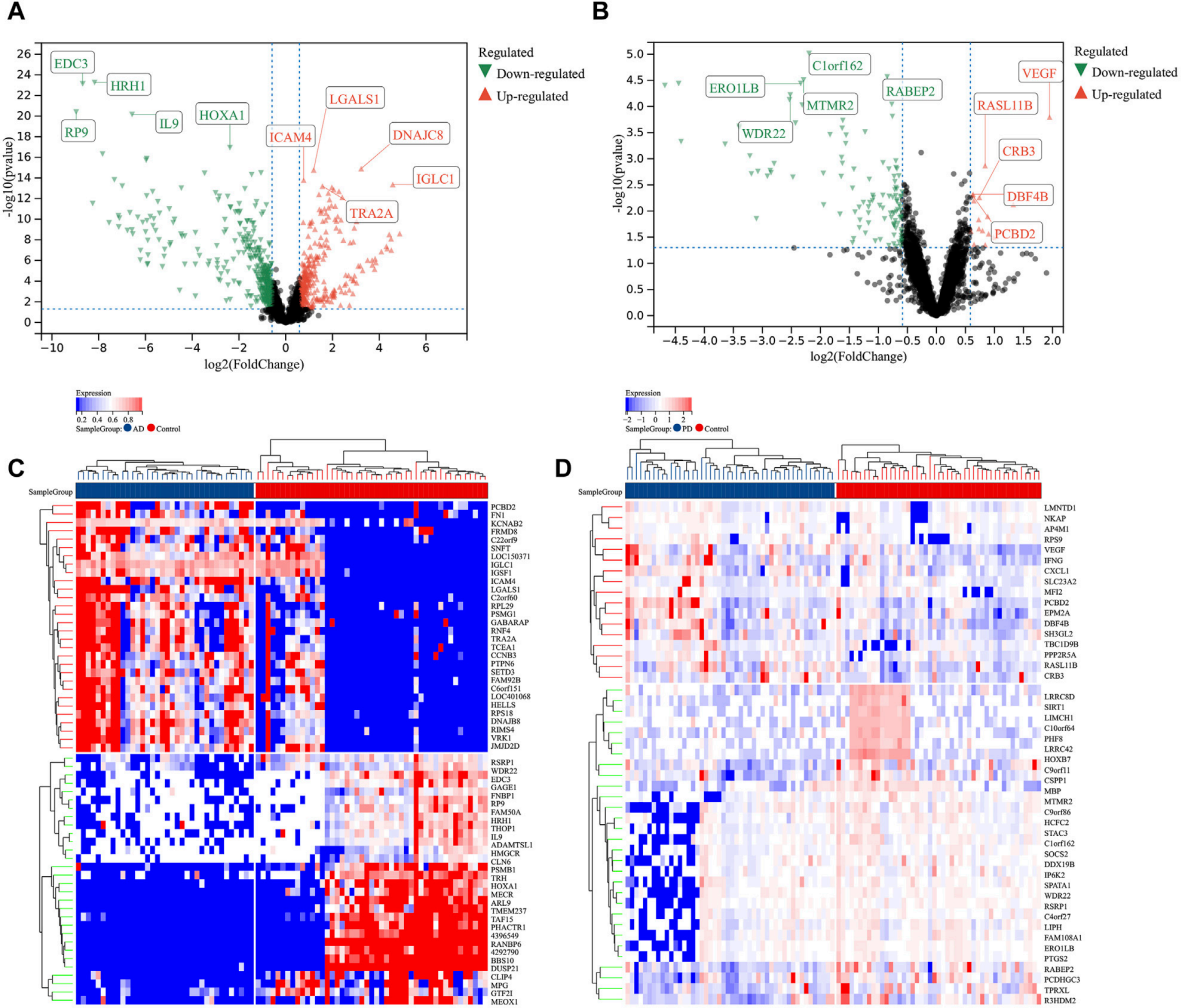
Identification of differentially expressed proteins (DEPs) in AD and PD. Volcano plots of DEPs in AD **(A)** and PD **(B)**. Heatmaps of the top 30 DEPs in AD **(C)** and PD **(D)**. Red, upregulated DEPs; blue, downregulated DEPs.

Human serum protein expression dataset of GSE39087 (AD and PD) was applied for our differential protein expression analysis. After data preprocessing and differential expression analysis, we identified 1988 DEPs between AD and healthy controls (|Fold change| ≥ 1.2 and *p*-value < 0.05), including 723 upregulated (DNAJC8, IGLC1, LGALS1, ICAM4, and TRA2A) and 1,265 downregulated proteins (EDC3, HRH1, RP9, IL9, and HOXA1) (Figure 2A). The top 30 DEPs of AD (Figure 2C) were visualized in heatmap. In addition, we also identified 344 DEPs between PD and healthy controls (|Fold change| ≥ 1.2 and *p*-value < 0.05), including 90 upregulated (VEGF, RASL11B, CRB3, DBF4B, and PCBD2) and 254 downregulated proteins (ERO1LB, C1orf162, WDR22, MTMR2, and RABEP2) (Figure 2B). And the top 30 DEPs of PD were visualized in heatmap (Figure 2D).

### WGCNA of the whole transcriptome expression matrix

**FIGURE 3:**
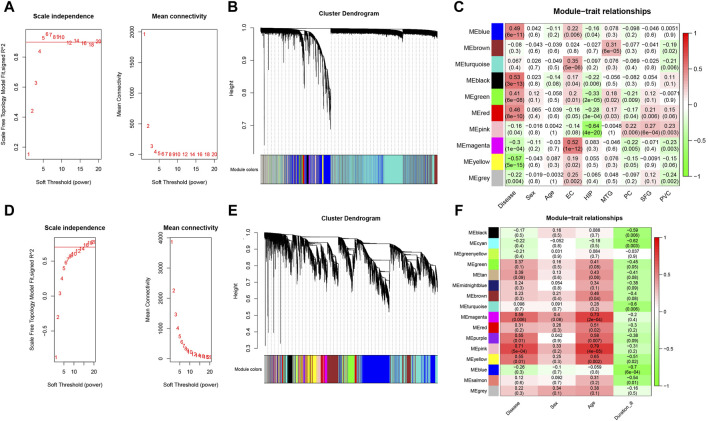
Identification of modules correlated with AD and PD in transcriptome datasets. Hierarchical clustering dendrograms were constructed for the samples in the AD **(A)** and PD **(B)** datasets. Additionally, module-trait relationship diagrams were generated for the AD **(C)** and PD **(D)** datasets. In these diagrams, each row represents a color module, while each column represents a clinical trait. The correlation and *p*-value for each module-trait combination are displayed in the respective cells. Furthermore, an analysis was performed to assess the scale independence and mean connectivity for the optimal soft threshold powers in the AD **(E)** and PD **(F)** datasets.

We performed WGCNA analysis to find similar gene expression patterns tended to exert similar biological functions in GSE5281 (AD) and GSE26927 (PD). Figures 3A, B displayed the hierarchical clustering dendrograms of the samples. Subsequently, we examined the soft threshold powers of the network topology and determined β values of 5 and 12 to be the optimal soft-thresholding parameters for GSE5281 (AD) and GSE26927 (PD) datasets (Figures 3E, F). From the GSE5281 dataset, we identified 9 modules (Figure 3C), while from the GSE26927 dataset, we identified 15 modules (Figure 3D). To investigate the associations between gene modules and AD or PD, we constructed module-trait diagrams. In GSE5281, the four modules with the highest correlation to AD were MEyellow (r = −0.57, *p* = 5e−15), MEblack (r = 0.53, *p* = 3e−13), MEblue (r = 0.49, *p* = 6e−11), and MEred (r = 0.46, *p* = 8e−10). In GSE26927, the four modules with the highest correlation to PD were MEpink (r = 0.71, *p* = 5e−04), MEmagenta (r = 0.59, *p* = 0.006), MEpurple (r = 0.55, *p* = 0.01), and MEyellow (r = 0.55, *p* = 0.01).

### Hypoxia-related genes involved in AD and PD shared by transcriptome and proteome

**FIGURE 4:**
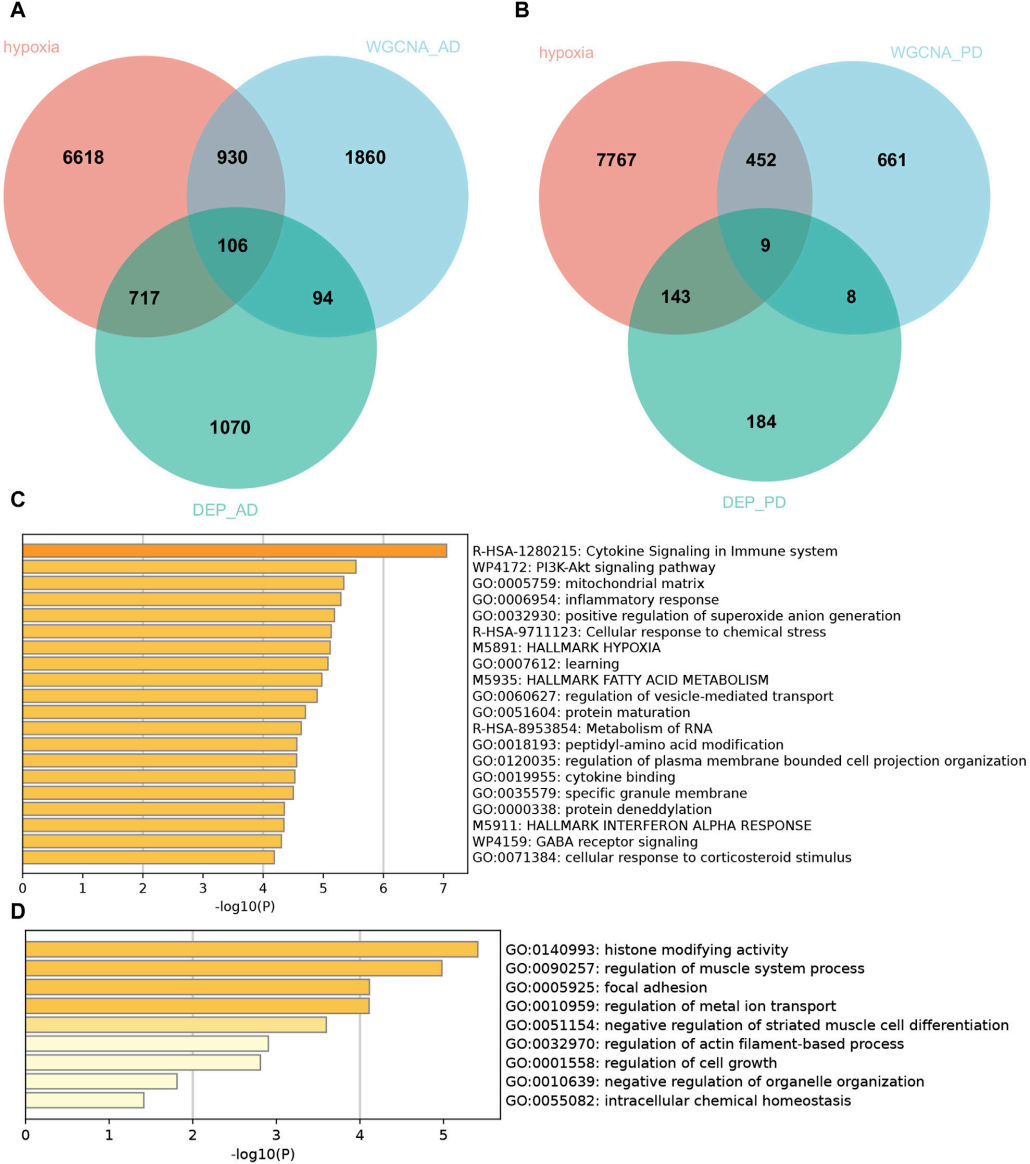
Hypoxia-related genes involved in AD and PD shared by transcriptome and proteome. Venn diagram of hypoxia-related genes shared by transcriptome and proteome in AD **(A)** and PD **(B)**. Pathway enrichment analyses of the common hypoxia-related genes in AD **(C)** and PD **(D)**.

To discover functional genes concurrently involved in both AD and PD across the transcriptome and proteome, we employed two methods to identify key genes. Firstly, we used WGCNA to identify modules that are related to AD and PD. Secondly, we conducted differential expression analysis to identify the most dysregulated genes. In total, we identified 200 genes in AD and 17 genes in PD that were concurrently involved in the transcriptome and proteome, respectively (Figures 4A, B). Enrichment analysis of concurrent Genes Involved in AD showed that the terms were mainly enriched in cytokine signaling in immune system, PI3K-Akt signaling pathway, mitochondrial matrix, inflammatory response, and positive regulation of superoxide anion generation (Figure 4C), whereas PD was related to histone modifying activity, regulation of muscle system process, focal adhesion, regulation of metal ion transport and negative regulation of striated muscle cell differentiation (Figure 4D).

In view of the important role of hypoxia-related genes in AD and PD, we extracted 106 and 9 hypoxia-related genes involved in AD and PD for further analysis by intersecting the DEPs and WGCNA related mRNAs with 8,371 hypoxia-related genes.

### Integrated development of AD and PD consensus signature

**FIGURE 5:**
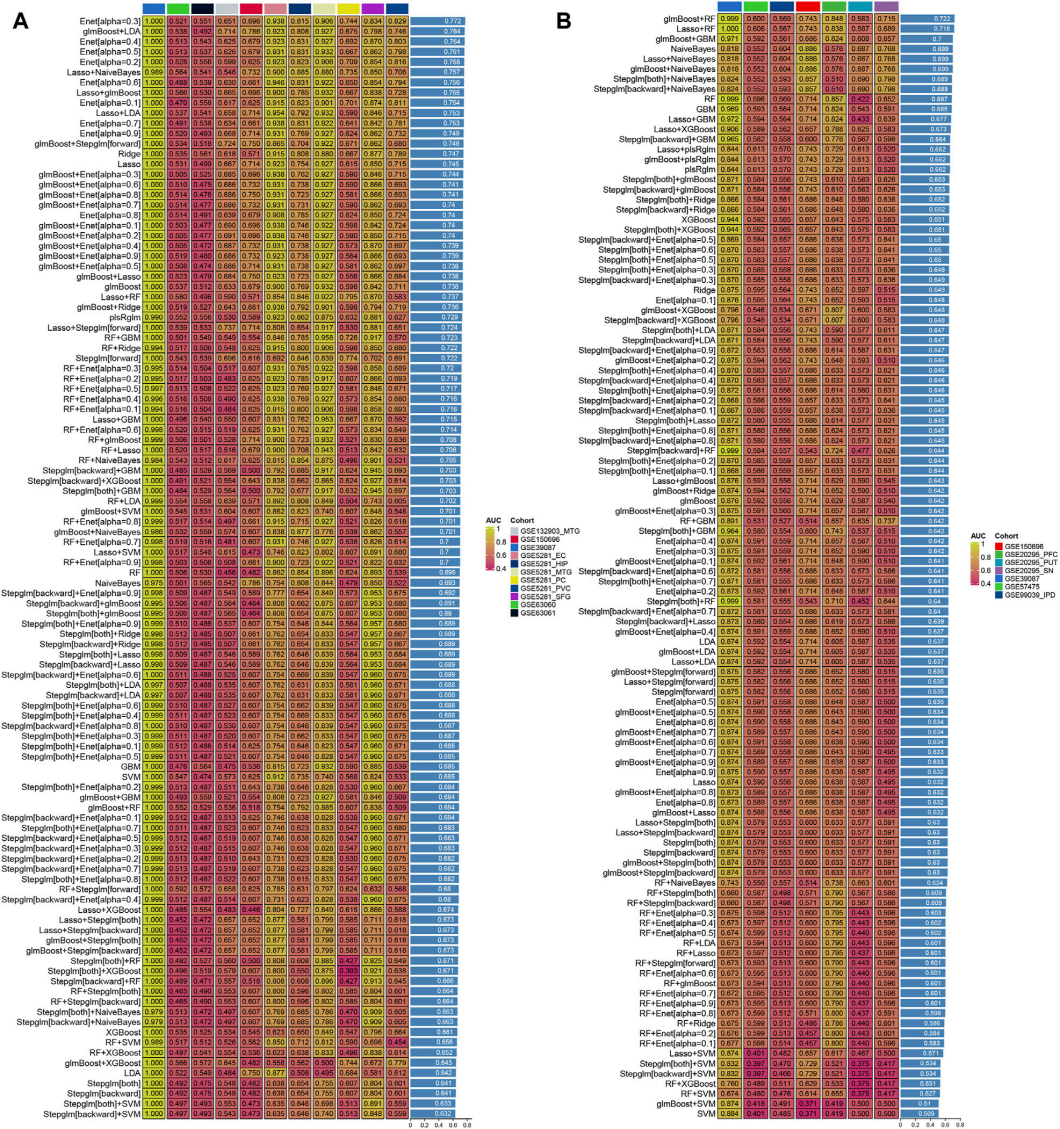
Generation of the artificial intelligence-derived hypoxia-related gene signature in AD and PD. The C-indices of 110 machine-learning algorithms in eleven cohorts of AD **(A)** and seven in PD **(B)**.

A total of 106 and 9 hypoxia-related genes involved in AD and PD were further incorporated into our integration program to develop an AIDHS. In the protein training cohort, we applied 110 algorithm combinations via ten-fold cross-validation to construct prediction models and calculated the average AUC of each algorithm in the remaining testing cohorts. In AD, the combination of glmBoost and LDA with higher average AUC (0.764) and proper genes (N = 17) was selected as the final model, which contained BLVRB, C10orf54, CDC37, CHCHD6, FKBPL, FN1, GLUL, HOXC4, PFN1, PPIL1, PRDX4, PSMB1, PSMG1, SLC16A14, SLC17A7, SMYD3, and SYK. In PD, the combination of glmBoost and RF with the highest average AUC (0.722) was selected as the final model (N = 3), including FMO5, HDAC4, and TGM2 (Figure 5).

### Altered of immune cell infiltration in AD and PD

**FIGURE 6:**
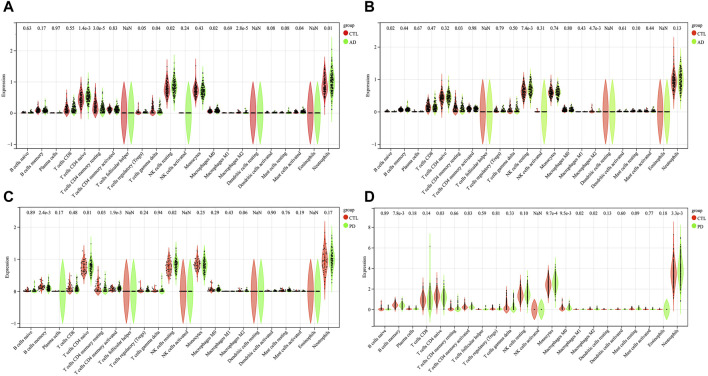
The results of immune cell infiltration analysis. A box plot of AD infiltrated immune cells in GSE60630 **(A)** and GSE60631 **(B)**. A box plot of PD infiltrated immune cells in GSE57475 **(C)** and GSE99039 **(D)**. *p < 0.05, **p < 0.01, ***p < 0.001, ****p < 0.0001.

We observed 8 (GSE60630) and 4 (GSE60631) significant differences in the infiltration of cells between AD and healthy samples. In GSE60630, T cells CD4 naïve, NK cells resting, Macrophages M0, Mast cells activated and Neutrophils cell types were upregulated, whereas T cells CD4 memory resting, T cells gamma delta and Macrophages M2 cell types downregulated (Figure 6A). In GSE60631, NK cells resting cell type was upregulated, whereas B cells naive, T cells CD4 memory resting and Macrophages M2 cell types downregulated (Figure 6B).

We observed 4 (GSE57475) and 7 (GSE99039) significant differences in the infiltration of cells between PD and healthy samples. In GSE57475, T cells CD4 memory activated and NK cells resting cell types were upregulated, whereas B cells memory and T cells CD4 memory resting cell types downregulated (Figure 6C). In GSE99039, 4 cell types were upregulated, including T cells CD4 naive, Monocytes, Macrophages M0, and Neutrophils; the three downregulated cell types were: B cells memory, Macrophages M1, and Macrophages M2 (Figure 6D).

### Correlation analysis between AIDHS markers and infiltrating cells

|r| > 0.2 was set as a correlation threshold for further analysis. In AD, T cells CD4 naïve cell was correlated with BLVRB, C10orf54, CDC37, CHCHD6, GLUL, HOXC4, and PPIL1 in GSE63060; and C10orf54, CHCHD6, GLUL, PFN1, PSMB1, and SYK in GSE63061. T cells CD4 memory resting cell was correlated with C10orf54, CDC37, GLUL, HOXC4, PPIL1, PRDX4, PSMB1, PSMG1, SLC16A14, and SYK in GSE63060; and BLVRB, C10orf54, CDC37, GLUL, HOXC4, PFN1, PPIL1, PRDX4, PSMB1, PSMG1, SLC17A7, and SYK in GSE63061. T cells gamma delta cell was correlated with C10orf54, PFN1, PPIL1, PRDX4, PSMB1, PSMG1, and SYK in GSE63060; and C10orf54, CHCHD6, GLUL, PFN1, PPIL1, PRDX4, PSMB1, PSMG1, and SYK in GSE63061. NK cells resting cell was correlated with BLVRB, CDC37, HOXC4, and PSMB1 in GSE63060; and C10orf54, CHCHD6, GLUL, HOXC4, PFN1, PRDX4, PSMB1, PSMG1, and SYK in GSE63061. Macrophages M0 cell was correlated with C10orf54, CHCHD6, GLUL, PPIL1, PSMG1, and SYK in GSE63060; and C10orf54, GLUL, PPIL1, PRDX4, PSMB1, PSMG1, and SYK in GSE63061.Macrophages M2 cell was correlated with C10orf54, PRDX4, PSMG1, and SYK in GSE63060; and GLUL in GSE63061. Mast cells activated cell was correlated with CDC37 and SMYD3 in GSE63060; and BLVRB, PPIL1, and SMYD3 in GSE63061. Neutrophils were correlated with C10orf54, CDC37, CHCHD6, GLUL, HOXC4, PPIL1, PRDX4, PSMB1, PSMG1, SMYD3, and SYK in GSE63060, and in GSE63061 (Figures 7A, B).

**FIGURE 7:**
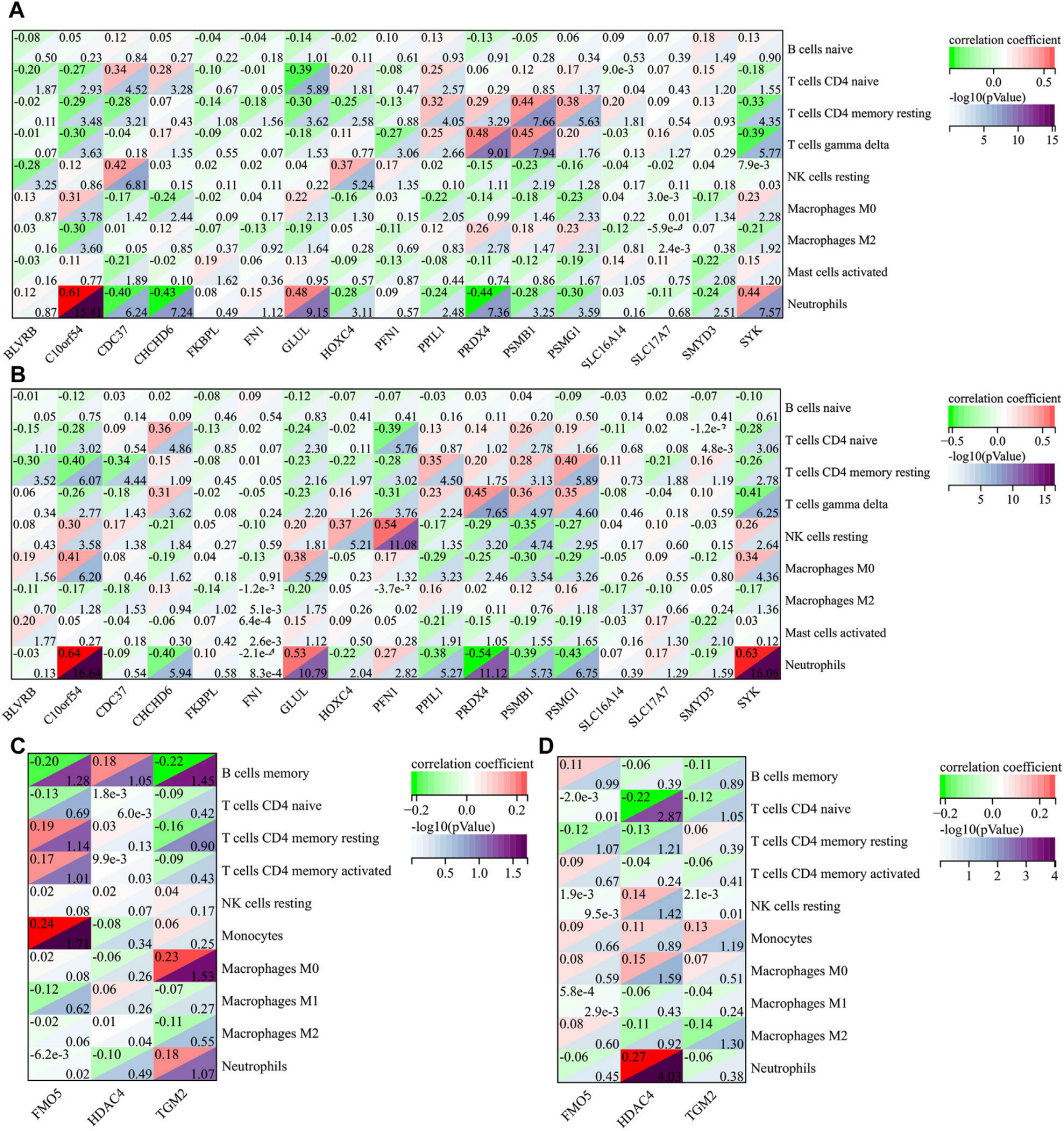
The correlation between AIDHS genes and infiltrated immune cells in AD (**(A)**: GSE60630, **(B)** GSE60630) and PD (**(C)**: GSE57475, **(D)** GSE99039). Nodes that appear redder indicate a stronger positive correlation, while nodes that appear bluer indicate a stronger negative correlation.

In PD, B cells memory cell was related to FMO5 and TGM2 in GSE57475. T cells CD4 naïve cell was related to HDAC4 in GSE99039. Monocytes was related to FMO5 in GSE57475. Macrophages M0 cell was related to TGM2 in GSE57475. Neutrophils was related to HDAC4 in GSE99039 (Figures 7C, D).

### TF–miRNA coregulatory network

**FIGURE 8:**
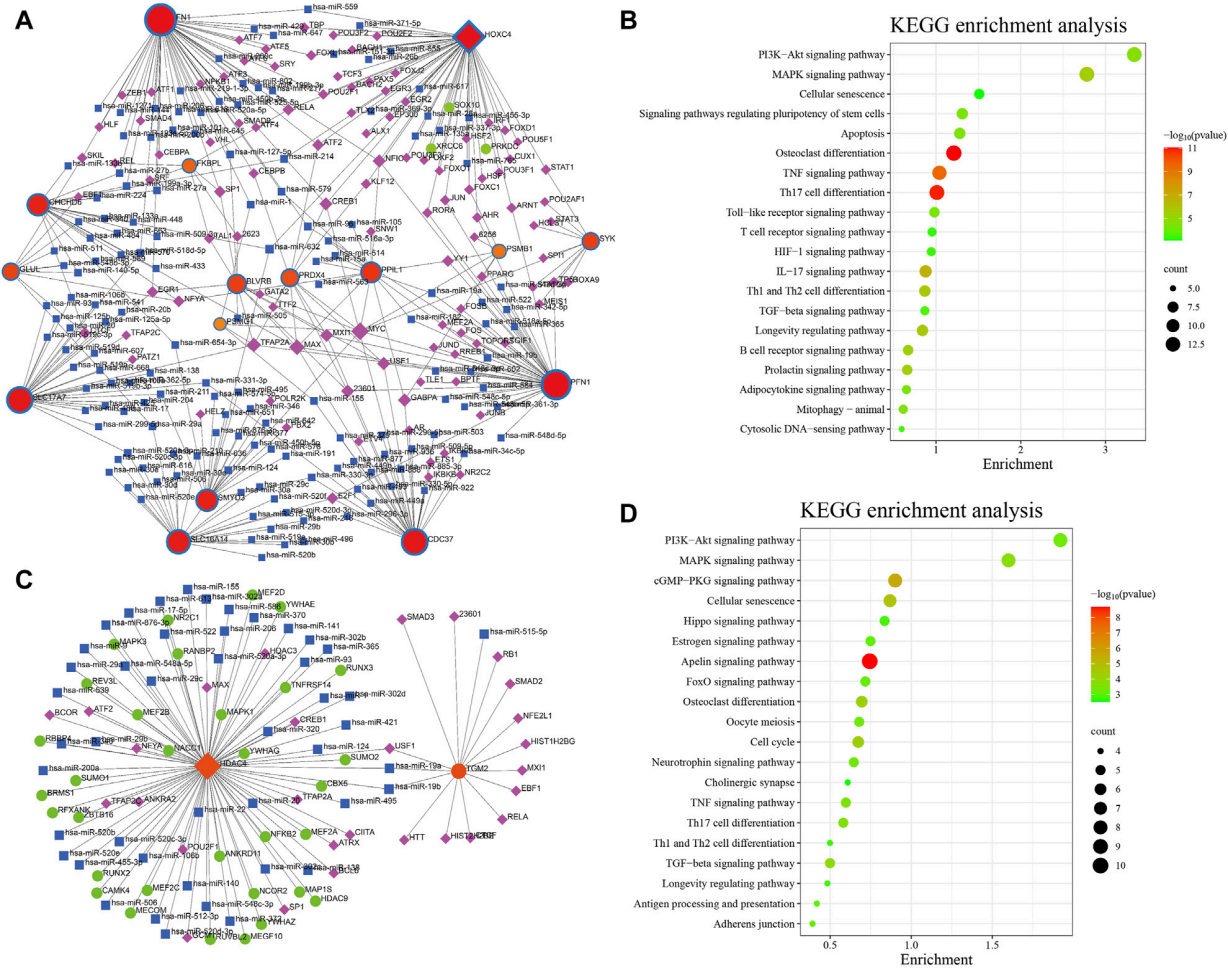
TF–miRNA coregulatory network and pathway enrichment analyses in AD **(A,B)** and PD **(C,D)**. The round nodes are AIDHS genes, the diamond nodes are TF genes, and the square nodes are miRNA genes.

The NetworkAnalyst online platform was used to generate a TF-miRNA co-regulatory network. The analysis of this network provided insights on the interaction between TFs, miRNAs, and the selected hub genes, which could explain the underlying mechanism of how hub gene expression is regulated. Figure 8A shows the TF–miRNA co-regulatory network. The AD TF–miRNA co-regulatory network is consisted of 269 nodes and 343 edges. A total of 149 miRNAs and 61 TF genes interacted with the validated AIDHS genes, including MYC, MAX, TFAP2A, USF1, NFIC, hsa-miR-632, hsa-miR-1, hsa-miR-124, hsa-miR-127-5p and hsa-miR-140-5p. Functional enrichment analysis showed these nodes were related to Osteoclast differentiation, Th17 cell differentiation, TNF signaling pathway, IL-17 signaling pathway, and Longevity regulating pathway (Figure 8B). HOXC4 owned the largest number of neighbors, followed by FN1, PFN1, CDC37, SLC17A7, SLC16A14, CHCHD6, SMYD3, PPIL1, BLVRB, and PRDX4.

Figure 8C shows the TF–miRNA co-regulatory network. The PD TF–miRNA co-regulatory network is consisted of 108 nodes and 109 edges. A total of 46 miRNAs and 28 TF genes interacted with the validated AIDHS genes, including USF1, TFAP2A, TFAP2C, SP1, POU2F1, hsa-miR-19a, hsa-miR-19b, hsa-miR-1, hsa-miR-106b and hsa-miR-124. Functional enrichment analysis showed these nodes were related to Apelin signaling pathway, cGMP-PKG signaling pathway, Cellular senescence, Cell cycle and Osteoclast differentiation (Figure 8D). HDAC4 owned the largest number of neighbors.

### Construct the signaling information network based on SIGNOR

**FIGURE 9:**
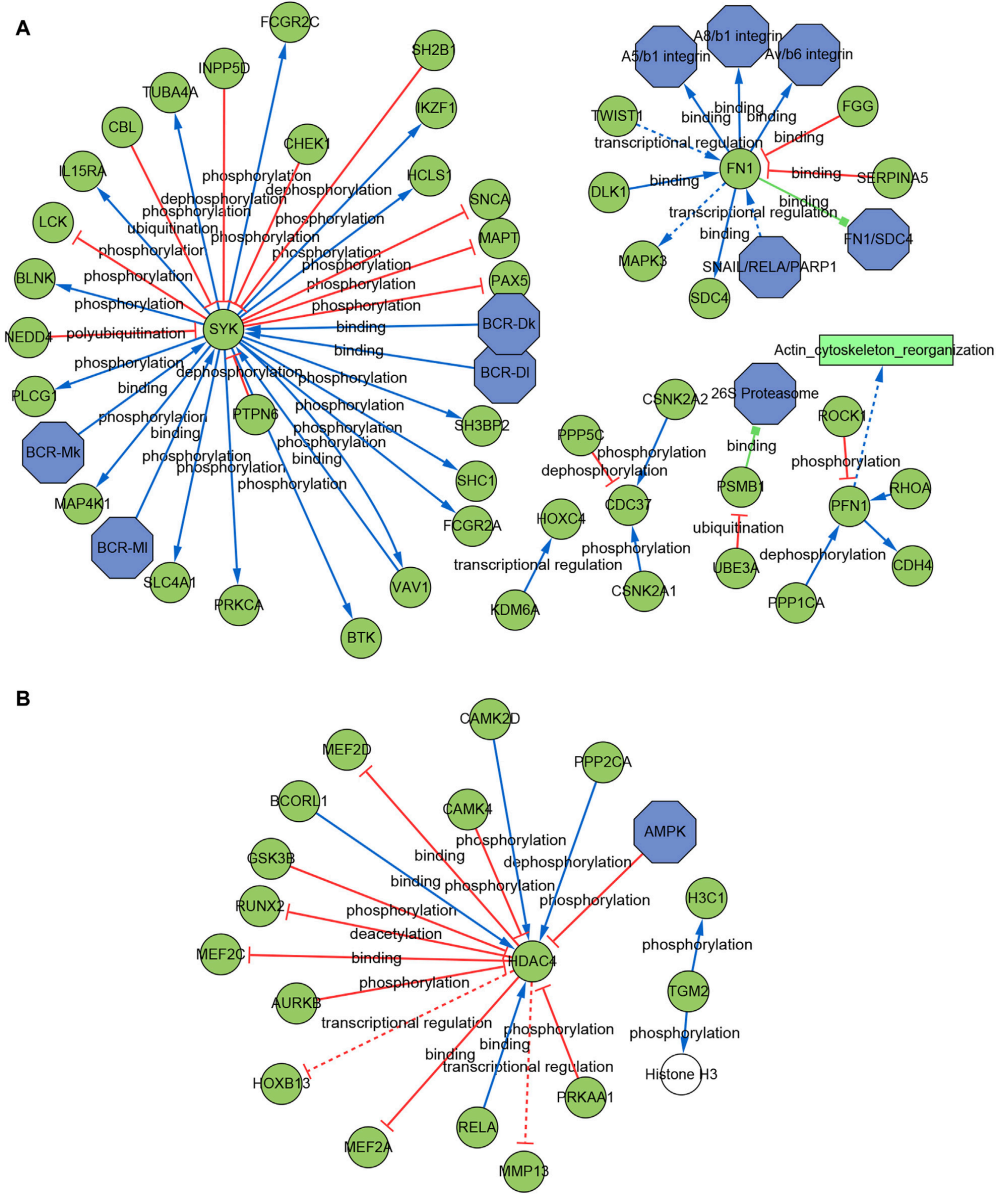
Signaling information network of the AIDHS genes through SIGNOR in AD **(A)** and PD **(B)**.

We further built the signaling information network of AIDHS through SIGNOR2.0. The AD signaling network included six interaction mechanisms, such as ubiquitination, transcriptional regulation, polyubiquitination, phosphorylation, dephosphorylation and binding. SYK showed the highest number of interaction, followed by FN1, PFN1, CDC37, and PSMB1. Moreover, FN1 interacted with more complex than other hub genes, including SNAIL/RELA/PARP1, FN1/SDC4, Av/b6 integrin, A8/b1 integrin, A5/b1 integrin complex. In addition, the analysis also identified the effect of actin cytoskeleton reorganization as potential phenotype (Figure 9A).

The PD signaling network included five interaction mechanisms, such as transcriptional regulation, phosphorylation, dephosphorylation, deacetylation and binding. HDAC4 showed the highest number of interaction and was inhibited by AMPK complex. TGM2 can directly phosphorylate H3C1 and Histone H3 (Figure 9B).

### Analysis the heterogeneity of striata in the AD and PD single-cell transcriptome

**FIGURE 10:**
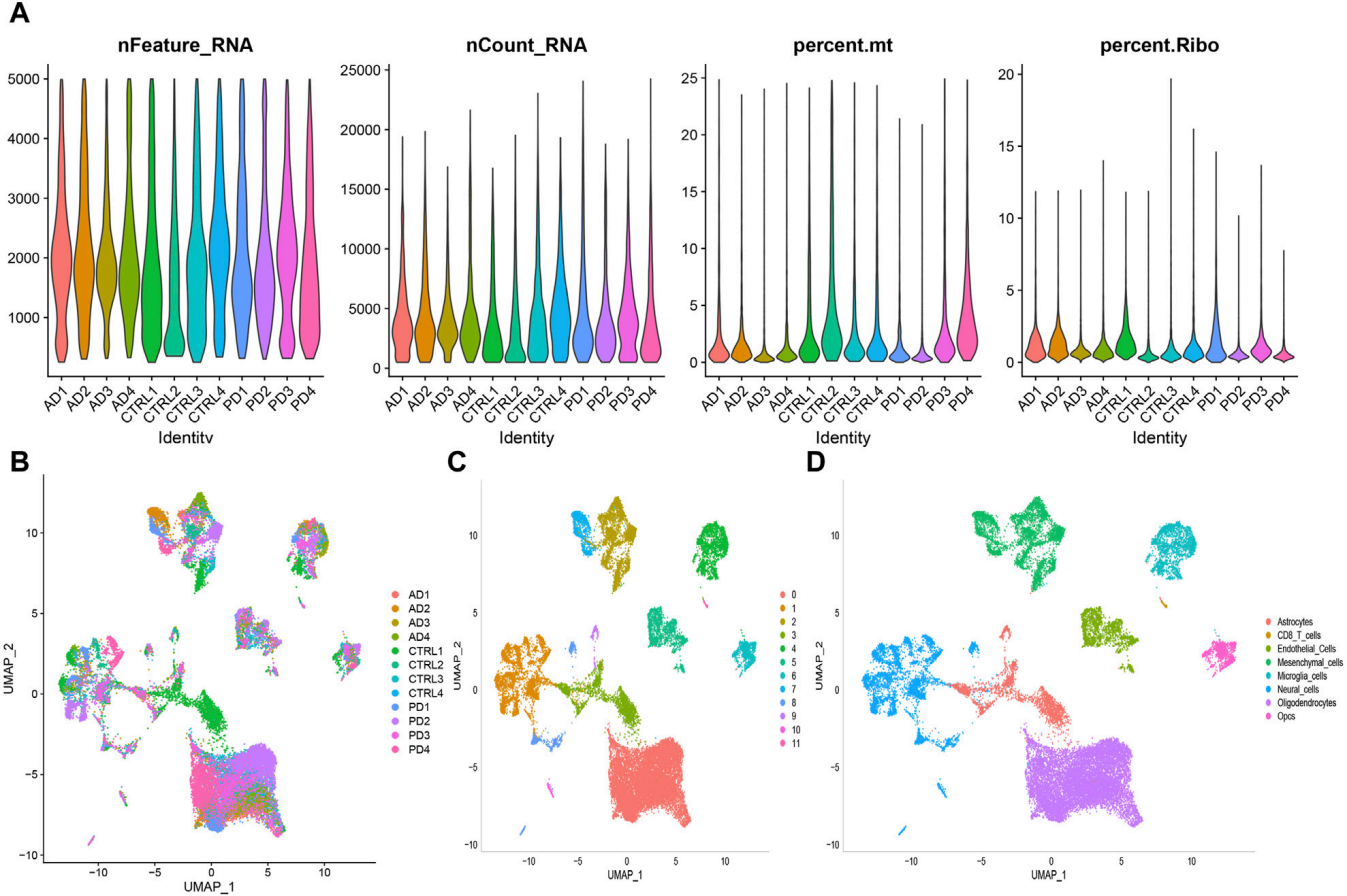
Preprocessing of scRNA-seq data was conducted to prepare for downstream analysis. **(A)** Following quality control steps, the counts, percentages, and features related to mitochondrial and ribosome genes were assessed for each sample under analysis. **(B)** Visualization of sample distribution was achieved using UMAP plots after integration of the datasets using the harmony algorithm. **(C)** A clustering algorithm with a resolution of 0.1 was applied, resulting in the identification of 12 clusters. **(D)** UMAP plot was used to show the distribution of the eight different cell types.

**FIGURE 11:**
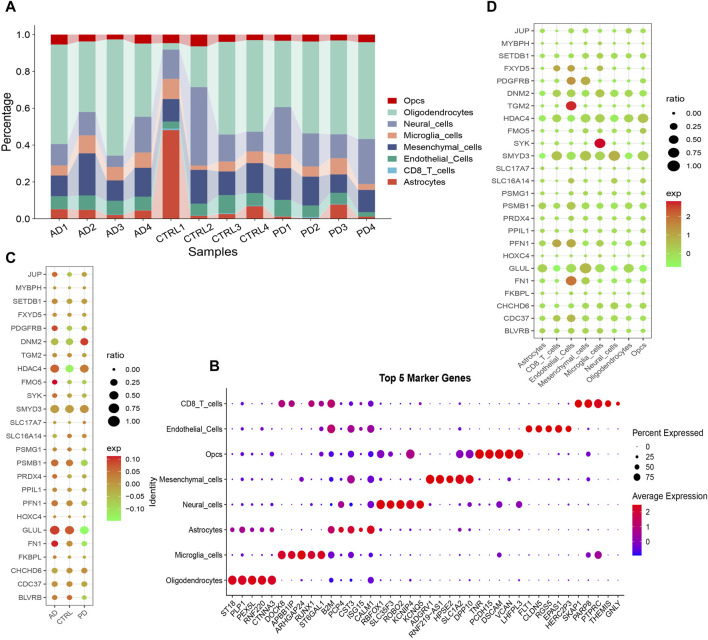
ScRNA-seq data was employed to annotate and scrutinize the distribution of cell proportions among the three groups. **(A)** The overall cell fraction comparison among the three groups. **(B)** A heatmap was produced to display the levels of expression of marker genes in the identified cells. Bubble plots depicting the expression of 26 AIDHS genes in different disease **(C)** and cell groups **(D)**.

The quality control summary of our single-cell RNA sequencing (scRNA-seq) data is presented in Figure 10A. After preprocessing the data, we used the harmony algorithm to merge the samples and effectively eliminate any potential batch effects. Figure 10B displays the representation of the merged dataset, which includes AD, PD and control samples after the implementation of the harmony algorithm. By following the standard procedures of Seurat, a total of 12 clusters were successfully identified and visualized using UMAP, as shown in Figure 10C. Subsequently, we observed eight distinct cell clusters (Figure 10D), including astrocytes (AQP4, GFAP), CD8 T cells (CCL5, NKG7, GZMK, GZMA, GZMM, CCL4, CD69, LGALS1, LGALS3, PDCD1, PRDM1, and CXCR6), endothelial cells (FLT1, CLDN5), mesenchymal cells (CD44, CHI3L1, and HIF1A), microglia cells (CSF1R, CD74, C3), neural cells (GAD1, GAD2, SOX2, and SLC17A7), oligodendrocytes (MBP, PLP1), and Opcs (PDGFRA, VCAN, CSPG4). Figure 11A showed the distribution of 8 cell types in 12 samples. And the top 5 marker genes were identified within the cell-type populations (Figure 11B). Figure 11C showed that most of the 26 AIDHS genes were differentially expressed among the three groups. These genes were specifically expressed in different cells, such as TGM2 being highly expressed in endothelial cells, PDGFRB being highly expressed in endothelial and mesenchymal cells, and SYK being highly expressed in microglia cells (Figure 11D).

### YXKFY restored the damaged SH-SY5Y cells caused by CoCl2 and reduce the ROS level

**FIGURE 12:**
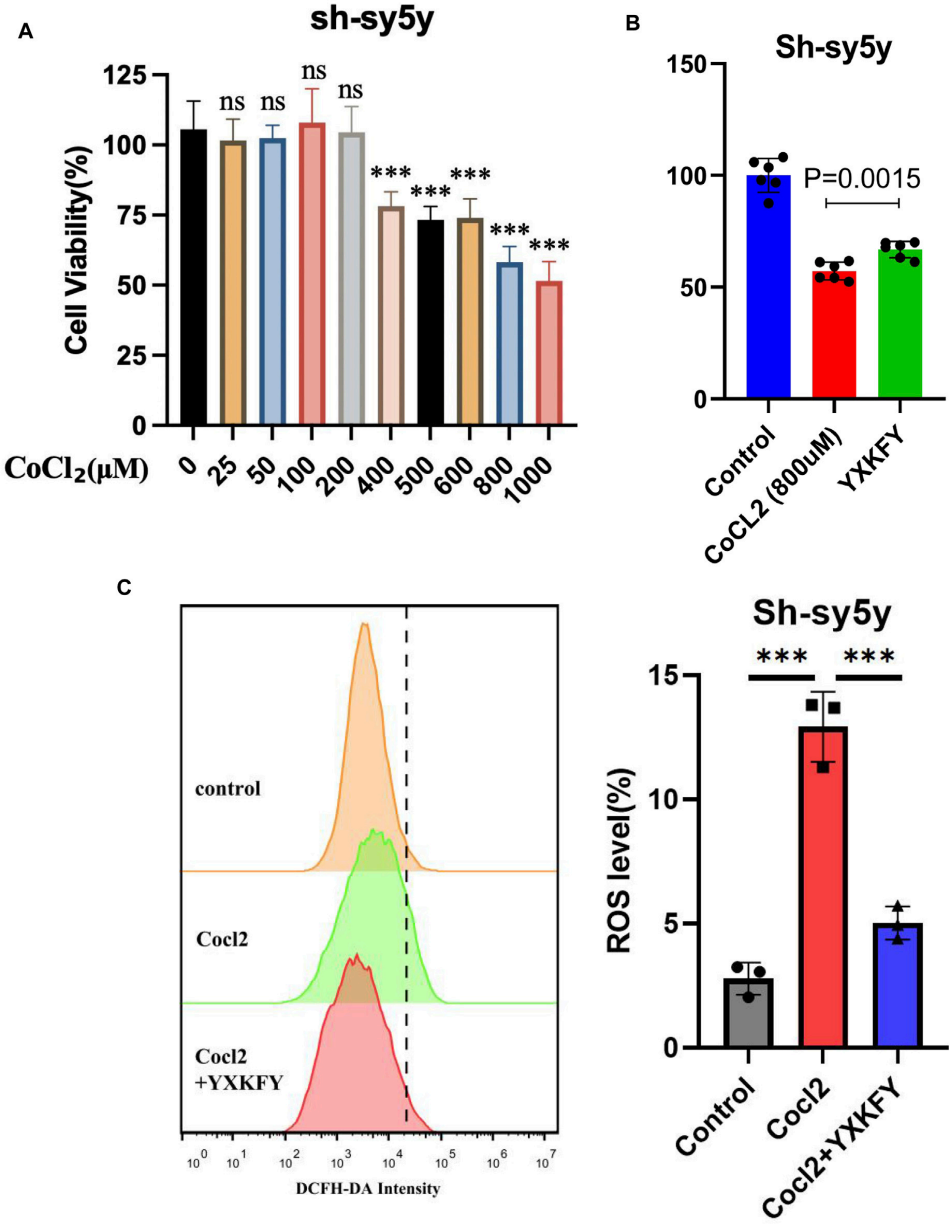
YXKFY restored the damaged SH-SY5Y cells caused by CoCl2 and reduce the ROS level. **(A)** The viability of SH-SY5Y cells was assessed using the CCK8 assay after exposure to different concentrations of CoCl2. **(B)** YXKFY-containing serum restored the damaged SH-SY5Y cells caused by CoCl2. **(C)** YXKFY-containing serum reduced the ROS level in the hypoxia model. ***: *p* < 0.001.

The viability of SH-SY5Y cells was assessed using the CCK8 assay after exposure to different concentrations of CoCl2 for 24 h. As depicted in Figure 12A, the viability of cells gradually declined as the concentration of CoCl2 increased from 400 to 1000 μM. Notably, when the CoCl2 concentration reached 800 μM, SH-SY5Y cell viability dropped to 57%. Subsequently, the concentration of 800 μM was selected for subsequent experiments as it induced significant damaging effects. In order to examine the potential of YXKFY in preventing CoCl2-induced cell death, the CCK8 assay was performed on SH-SY5Y cells treated with YXKFY-containing serum in the presence of CoCl2. As depicted in Figure 12B, the addition of YXKFY-containing serum led to a notable improvement in cell viability, with an increase to 67%. Moreover, YXKFY-containing serum can reduce the ROS level in the hypoxia model (Figure 12C).

### Validation of hub genes

**FIGURE 13:**
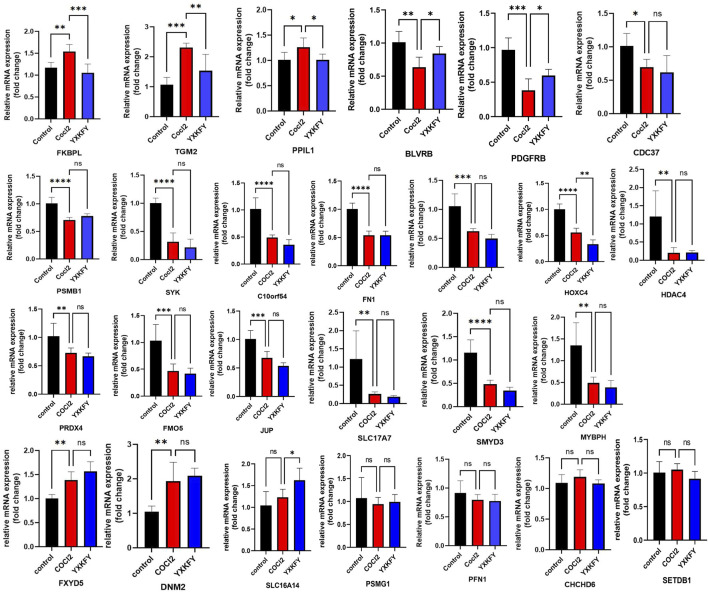
Validation of the mRNA expression levels of AIDHS genes. *: *p* < 0.05, **: *p* < 0.01, ***: *p* < 0.001.

In order to validate the findings of the bioinformatics analysis, we identified the 17 AIDHS genes of AD and 9 AIDHS genes of PD. The mRNA expression levels of these genes were assessed using RT-qPCR. The hypoxia group exhibited decreased expression of BLVRB, C10orf54, CDC37, FKBPL, FN1, GLUL, HOXC4, PPIL1, PRDX4, PSMB1, SLC17A7, SMYD3, SYK, FMO5, HDAC4, TGM2, PDGFRB, MYBPH, and JUP and increased expression of FXYD5 and DNM2 compared to the control group. Additionally, the expression of FKBPL, TGM2, PPIL1, BLVRB, and PDGFRB showed substantial recovery after YXKFY treatment (Figure 13).

### Identification of the components in YXKFY and YXKFY-containing serum

Based on the information provided by high-resolution mass spectrometry, such as accurate molecular masses for quasi-molecular ions and loading ions, the precise relative molecular mass of the primary mass spectra is inferred. The preliminary estimation of the various components obtained under high-resolution mass spectrometry is then conducted using the Peakview 1.2 software for molecular formula fitting. The HPLC analysis identified a total of 107 chemical components in the YXKFY, and a further 9 prototype components were identified in the YXKFY-containing serum (Table 2).

**TABLE 2:** The detailed information of chemical components derived from YXKFY by UPLC-Q/TOF-MS.

No.	Rt/min	Ion mode	Measured mass/Da	Molecular formula	Calculated mass/Da	Error/ppm	Identification	Peak area	Peak Area_Plasma
1	0.912	[M + H]^+^	183.0858	C6H15O6	183.0863	−2.8	Sorbitol	538,380	0
2	0.921	[M-H]^-^	179.0570	C6H11O6	179.0561	5.0	Glucose	1,676,539	0
3	0.996	[M-H]^-^	191.0566	C7H11O6	191.0561	2.6	Quinic acid	3,460,700	7,512
4	0.999	[M + H]^+^	116.0698	C5H10NO2	116.0706	−6.9	Proline	575,886	0
5	1.009	[M-H]^-^	341.1085	C12H21O11	341.1089	−1.3	Sucrose	3,205,474	0
6	1.122	[M-H]^-^	133.0147	C4H5O5	133.0143	3.4	Succinic acid	51,667	0
7	1.240	[M + H]^+^	171.0285	C7H7O5	171.0288	−1.8	Gallic acid	173,573	0
8	1.355	[M + H]^+^	155.0335	C7H7O4	155.0339	−2.5	Protocatechuic acid isomer	202,678	0
9	1.443	[M-H]^-^	191.0204	C6H7O7	191.0197	3.5	Citric acid	989,583	0
10	1.451	[M + H]^+^	442.1446	C19H20N7O6	442.1470	−5.3	Folcidin	20,070	0
11	1.732	[M + H]^+^	245.0759	C9H13N2O6	245.0768	−3.7	Uridine	87,619	0
12	1.735	[M + H]^+^	113.0339	C4H5N2O2	113.0346	−5.8	Uracil	45,669	0
13	1.800	[M + H]^+^	170.0809	C8H12NO3	170.0812	−1.6	Pyridoxine	344,564	0
14	2.119	[M + H]^+^	132.1009	C6H14NO2	132.1019	−7.6	Leucine	50,928	0
15	2.179	[M-H]^-^	169.0148	C7H5O5	169.0143	3.3	Gallic acid isomer	9,768	0
16	2.275	[M-H]^-^	375.1293	C17H19N4O6	375.1310	−4.6	Riboflavine isomer	2,690	0
17	2.297	[M + H]^+^	132.1015	C6H14NO2	132.1019	−3.1	Leucine isomer	53,312	0
18	2.346	[M + H]^+^	268.1042	C10H14N5O4	268.1040	0.6	Adenosine	41,479	0
19	2.497	[M + H]^+^	180.1014	C10H14NO2	180.1019	−2.8	Maltoxazine isomer	14,685	0
20	2.507	[M + H]^+^	132.1009	C6H14NO2	132.1019	−7.6	Leucine isomer	3,040	0
21	2.553	[M + H]^+^	144.1020	C7H14NO2	144.1019	0.7	Stachydrine	43,600	0
22	2.795	[M-H]^-^	169.0148	C7H5O5	169.0143	3.3	Gallic acid isomer	3,642	0
23	2.860	[M + H]^+^	121.0638	C8H9O	121.0648	−8.2	Acetophenone isomer	6,696	0
24	2.927	[M + H]^+^	180.1017	C10H14NO2	180.1019	−1.1	Maltoxazine	208,124	0
25	3.266	[M-H]^-^	375.1289	C17H19N4O6	375.1310	−5.6	Riboflavine isomer	3,815	0
26	4.368	[M + H]^+^	139.0387	C7H7O3	139.0390	−1.9	p-Hydroxybenzoic acid	8,603	0
27	4.580	[M-H]^-^	153.0199	C7H5O4	153.0193	3.7	3,4-Dihydroxybenzoic acid	53,048	0
28	6.523	[M + H]^+^	137.0594	C8H9O2	137.0597	−2.2	4′-Hydroxyacetophenone	31,247	0
29	7.179	[M + H]^+^	169.0490	C8H9O4	169.0495	−3.2	Vanillic acid isomer	14,596	0
30	7.569	[M-H]^-^	137.0251	C7H5O3	137.0244	5.0	p-Hydroxybenzoic acid isomer	16,430	0
31	7.976	[M-H]^-^	153.0201	C7H5O4	153.0193	5.0	Protocatechuic acid	57,954	0
32	8.002	[M + H]^+^	155.0332	C7H7O4	155.0339	−4.4	Protocatechuic acid isomer	31,047	0
33	8.105	[M + H]^+^	139.0384	C7H7O3	139.0390	−4.1	p-Hydroxybenzoic acid isomer	17,940	0
34	8.169	[M + H]^+^	355.1010	C16H19O9	355.1024	−3.8	Chlorogenic acid	12,483	0
35	8.474	[M + H]^+^	146.1168	C7H16NO2	146.1176	−5.2	Acetylcholine	3,356	0
36	8.514	[M + H]^+^	193.0494	C10H9O4	193.0495	−0.7	Scopoletin	86,514	0
37	10.068	[M + H]^+^	220.1182	C9H18NO5	220.1180	1.1	Vitamin B5	2,572	0
38	11.137	[M-H]^-^	153.0193	C7H5O4	153.0193	−0.2	Protocatechuic acid isomer	5,234	0
39	11.615	[M-H]^-^	137.0248	C7H5O3	137.0244	2.8	p-Hydroxybenzoic acid isomer	9,789	0
40	12.202	[M + H]^+^	169.0489	C8H9O4	169.0495	−3.8	Vanillic acid	12,655	0
41	12.733	[M + H]^+^	355.1023	C16H19O9	355.1024	−0.2	Neochlorogenic acid	106,958	0
42	13.679	[M + H]^+^	139.0384	C7H7O3	139.0390	−4.1	p-Hydroxybenzoic acid isomer	6,711	0
43	13.794	[M + H]^+^	265.1434	C15H21O4	265.1434	−0.1	Tauremisin	7,131	0
44	13.817	[M + H]^+^	355.1016	C16H19O9	355.1024	−2.1	Chlorogenic acid isomer	78,977	0
45	13.927	[M + H]^+^	328.1533	C19H22NO4	328.1543	−3.2	Stepholidine	11,349	0
46	14.065	[M + H]^+^	195.0649	C10H11O4	195.0652	−1.5	Ferulic acid	20,975	0
47	14.066	[M + H]^+^	107.0485	C7H7O	107.0491	−6.0	Benzaldehyde isomer	8,745	0
48	14.336	[M + H]^+^	193.0491	C10H9O4	193.0495	−2.3	Scopoletin isomer	15,683	0
49	14.466	[M + H]^+^	199.0595	C9H11O5	199.0601	−3.0	Syringic acid	9,747	0
50	14.590	[M-H]^-^	431.1544	C19H27O11	431.1559	−3.4	Zizybeoside I	21,089	0
51	14.668	[M + H]^+^	286.1437	C17H20NO3	286.1438	−0.2	Coclaurine	362,560	0
52	14.839	[M + H]^+^	298.1437	C18H20NO3	298.1438	−0.2	Lysicamine	20,761	293
53	15.304	[M-H]^-^	375.1283	C17H19N4O6	375.1310	−7.2	Riboflavine	7,526	550
54	15.384	[M + H]^+^	151.0748	C9H11O2	151.0754	−3.7	Benzyl acetate	39,685	0
55	15.481	[M + H]^+^	579.1688	C27H31O14	579.1708	−3.5	Vitexin 2″-O-rhamnoside isomer	5,046	0
56	15.598	[M-H]^-^	593.2068	C25H37O16	593.2087	−3.2	Zizybeoside Ⅱ	6,509	0
57	15.667	[M + H]^+^	433.1689	C19H29O11	433.1704	−3.6	Zizybeoside I isomer	11,773	0
58	15.737	[M + H]^+^	149.0596	C9H9O2	149.0597	−0.7	Cinnamic acid	138,605	3,943
59	15.738	[M + H]^+^	121.0646	C8H9O	121.0648	−1.6	Acetophenone	25,054	0
60	16.187	[M + H]^+^	757.2143	C33H41O20	757.2186	−5.6	Kaempferol 3-sophoroside-7-rhamnoside isomer	1,508	0
61	16.312	[M + H]^+^	265.1435	C15H21O4	265.1434	0.2	Tauremisin isomer	5,990	0
62	16.365	[M + H]^+^	387.2005	C19H31O8	387.2013	−2.2	Roseoside	34,972	0
63	16.366	[M + H]^+^	225.1481	C13H21O3	225.1485	−1.9	Vomifoliol	12,846	0
64	16.871	[M + H]^+^	377.1445	C17H18NO2	377.1456	−2.8	Vitamin B2	10,101	0
65	16.944	[M + H]^+^	757.2171	C33H41O20	757.2186	−1.9	Kaempferol 3-sophoroside-7-rhamnoside isomer	2,400	0
66	17.088	[M + H]^+^	387.2003	C19H31O8	387.2013	−2.7	Roseoside isomer	7,682	0
67	17.381	[M + H]^+^	757.2161	C33H41O20	757.2186	−3.3	Kaempferol 3-sophoroside-7-rhamnoside isomer	3,779	0
68	17.912	[M + H]^+^	449.1073	C21H21O11	449.1078	−1.2	Astragalin	48,278	0
69	18.184	[M + H]^+^	268.1320	C17H18NO2	268.1332	−4.5	Asimilobine	29,885	0
70	18.366	[M + H]^+^	287.0525	C15H11O6	287.0550	−8.8	Kaempferol	10,734	0
71	18.393	[M + H]^+^	757.2205	C33H41O20	757.2186	2.5	Kaempferol 3-sophoroside-7-rhamnoside isomer	1901	0
72	18.613	[M-H]^-^	137.0250	C7H5O3	137.0244	4.2	p-Hydroxybenzoic acid	88,834	40,444
73	18.616	[M + H]^+^	757.2158	C33H41O20	757.2186	−3.7	Kaempferol 3-sophoroside-7-rhamnoside	1,245	0
74	18.660	[M-H]^-^	609.1445	C27H29O16	609.1461	−2.6	Quercetin 3-O-rutinoside	2,246	0
75	18.828	[M + H]^+^	579.1693	C27H31O14	579.1708	−2.6	Vitexin 2″-O-rhamnoside	12,438	0
76	18.981	[M + H]^+^	193.0492	C10H9O4	193.0495	−1.7	Scopoletin isomer	23,891	0
77	19.026	[M + H]^+^	433.1112	C21H21O10	433.1129	−4.0	Kaempferol 7-O-rhamnoside	7,534	0
78	19.149	[M-H]^-^	577.1547	C27H29O14	577.1563	−2.7	Isovitexin 2″-O-rhamnoside	10,637	0
79	19.346	[M + H]^+^	579.1697	C27H31O14	579.1708	−2.0	Vitexin 2″-O-rhamnoside isomer	35,547	0
80	19.658	[M + H]^+^	449.1066	C21H21O11	449.1078	−2.8	Quercetin-7-O-α-L-Rhamnoside	33,657	3,377
81	19.686	[M-H]^-^	609.1454	C27H29O16	609.1461	−1.2	Quercetin 3-glucoside 7-rhamnoside	1,648	0
82	19.806	[M + H]^+^	449.1070	C21H21O11	449.1078	−1.9	Astragalin isomer	33,234	0
83	19.851	[M-H]^-^	609.1453	C27H29O16	609.1461	−1.3	Rutin	7,175	0
84	20.070	[M + H]^+^	625.1749	C28H33O16	625.1763	−2.3	Isorhamnetin-3-O-rutinoside	38,081	0
85	21.330	[M + H]^+^	479.1157	C22H23O12	479.1184	−5.6	Isorhamnetin-3-O-glucoside	5,304	0
86	21.594	[M + H]^+^	282.1493	C18H20NO2	282.1489	1.6	Nornuciferine	43,012	0
87	21.608	[M + H]^+^	625.1751	C28H33O16	625.1763	−1.9	Narcissin	25,240	0
88	21.810	[M + H]^+^	479.1172	C22H23O12	479.1184	−2.5	Isorhamnetin 3-O-galactoside	8,510	0
89	21.899	[M + H]^+^	165.0907	C10H13O2	165.0910	−1.9	Eugenol	35,879	0
90	22.875	[M + H]^+^	247.1324	C15H19O3	247.1329	−1.9	Zederone	10,755	0
91	23.949	[M + H]^+^	291.0861	C15H15O6	291.0863	−0.7	Catechin	21,686	0
92	24.750	[M + H]^+^	219.1743	C15H23O	219.1743	−0.2	Tumerone	77,472	0
93	24.916	[M + H]^+^	433.1123	C21H21O10	433.1129	−1.4	Vitexin	4,581	0
94	25.676	[M + H]^+^	115.1110	C7H15O	115.1117	−6.4	Heptanal isomer	8,247	0
95	27.283	[M + H]^+^	107.0485	C7H7O	107.0491	−6.0	Benzaldehyde	36,091	0
96	27.744	[M + H]^+^	173.1533	C10H21O2	173.1536	−1.8	Isoamyl isovalerate	34,456	0
97	27.751	[M + H]^+^	115.1111	C7H15O	115.1117	−5.6	Heptanal	26,140	0
98	28.044	[M + H]^+^	219.1736	C15H23O	219.1743	−3.4	Tumerone	8,308	7,917
99	29.112	[M + H]^+^	487.3401	C30H47O5	487.3418	−3.5	Ceanothic acid	8,080	0
100	29.453	[M-H]^-^	157.1239	C9H17O2	157.1234	3.2	Ethyl heptanoate	11,742	0
101	29.631	[M + H]^+^	159.1375	C9H19O2	159.1380	−2.9	Propionyl hexanoate	10,372	0
102	29.805	[M-H]^-^	169.1238	C10H17O2	169.1234	2.3	Isoamyl Senecioate	1878	1,256
103	30.270	[M + H]^+^	229.0854	C14H13O3	229.0859	−2.3	trans-Resveratrol	16,971	0
104	30.591	[M + H]^+^	219.1739	C15H23O	219.1743	−2.0	Tumerone	13,849	20,300
105	32.050	[M + H]^+^	235.1688	C15H23O2	235.1693	−1.9	Curcumenol	175,822	0
106	38.723	[M + H]^+^	135.0802	C9H11O	135.0804	−1.8	Chavicol	83,475	0
107	42.510	[M + H]^+^	257.2473	C16H33O2	257.2475	−0.8	Palmitic acid	272,046	0

### Molecular docking validation

The present research investigated binding affinity between the above five genes and the 8 prototype ingredients in the YXKFY-containing serum. The molecular docking results revealed different levels of binding between natural products and hub genes, with the majority of natural products showing good binding capabilities to the hub genes. Particularly, riboflavin and lysicamine exhibited higher binding affinity than other compounds (Table 3).

**TABLE 3:** The protein-ligand binding energy of molecular docking results (kcal/mol).

Ligand	BLVRB (5oog)	FKBPL (AlphaFold)	PDGFRB (AlphaFold)	PPIL1 (2 × 7k)	TGM2 (3s3j)
Cinnamic acid	−5.7	−5.4	−5.2	−5.8	−5.8
Isoamyl Senecioate	−5.1	−4.7	−4.1	−4.9	−5.3
Lysicamine	−7.5	−7.2	−6.4	−7.4	−7.1
p-Hydroxybenzoic acid	−5.3	−5	−4.7	−5.7	−5.5
Quinic acid	−5.4	−5.2	−4.6	−6.1	−5.6
Riboflavin	−7.6	−6.6	−6.3	−8.2	−7.5
Tumerone	−6.6	−5.9	−5.5	−5.5	−6.7

## Discussion

To this day, researchers continue to face the challenging task of finding an effective drug for treating AD and PD using modern medicine. However, there is a growing body of evidence suggesting that traditional Chinese medicine may offer promise in the prevention and treatment of AD and PD (Pei et al., 2020; Chen et al., 2022). Hypoxia, characterized by oxygen deprivation, is implicated in neuronal stress, initiating a cascade that culminates in neuronal dysfunction and demise, thereby exacerbating the pathogenesis and progression of AD and PD (March-Diaz et al., 2021; Guo et al., 2022). Our study, therefore, presents an integrative strategy, merging bidirectional transcriptome and proteome analyses, to delineate the hypoxia-related molecular signatures and pathways that underpin the neuroprotective efficacy of YXKFY in both AD and PD.

In our study, we first profiled hypoxia-related mRNA and protein expression in AD and PD. Our analysis revealed that the dysregulated mRNAs and proteins in both conditions predominantly converge on mitochondrial and inflammatory pathways in AD, and pathways related to the muscle system in PD. It is a recognized phenomenon that hypoxia triggers the activation of multiple signaling cascades, notably cytokine signaling within the immune system (Sturrock et al., 2018) and the PI3K-Akt pathway (Pan et al., 2021), which are integral to inflammatory responses and the modulation of mitochondrial function (Wang et al., 2021). The mitochondrial matrix is acutely vulnerable to hypoxia, which can augment the production of superoxide anions, thereby exacerbating oxidative stress and neuroinflammation. (Spooner et al., 2021). Furthermore, hypoxia has been implicated in the upregulation of pro-inflammatory cytokines and chemokines, perpetuating the chronic inflammatory milieu characteristic of AD and PD (Merelli et al., 2021). Additionally, hypoxia impacts muscle function, with disruptions in muscle system processes linked to the motor symptoms of PD (Miterko et al., 2021). Consequently, elucidating the molecular underpinnings of hypoxia’s impact on these pathways is essential for devising innovative therapeutic strategies for both AD and PD.

In this study, we conducted a comprehensive analysis using nine independent cohorts to identify stable AIDHS for the development of AD and PD. Recognizing the potential influence of researcher preferences and biases in algorithm selection, we employed a rigorous approach by integrating ten distinct machine learning algorithms to discern the optimal signature from a comprehensive set of 113 algorithmic combinations. This strategy culminated in the identification of AIDHS in AD (17 genes) and PD (3 genes). Our *in vitro* validation corroborated the machine learning predictions, with the majority of genes showing significant differential expression following hypoxia treatment. Notably, YXKFY was found to ameliorate the dysfunction of five key genes (FKBPL, TGM2, PPIL1, BLVRB, and PDGFRB). While some of the biomarkers associated with these genes have been previously implicated in AD and PD, others represent novel findings in the context of neurodegenerative diseases. PPIL1 plays a crucial role as an enzyme-substrate pair within the spliceosome, exerting its function in the facilitation of RNA splicing and ensuring the survival of neurons, and its mutations may cause neurodegenerative disease (Chai et al., 2021). The silencing of TGM2, implicated in the pathogenesis of AD and PD, inhibited mitochondrial calcium influx, accumulation of mtROS, phosphorylation of Tau, and ultimately protected neuronal cells from Aβ-induced cell death (Lee et al., 2021). The depletion of PDGFRB within the precuneus region in individuals with AD has been linked to fibrinogen leakage, a decline in oxygenation, and the accumulation of fibrillar Aβ (Miners et al., 2018). Therefore, PPIL1, TGM2 and PDGFRB may serve as therapeutic targets of YXKFY in AD and PD. However, FKBPL and BLVRB have never been investigated in AD and PD, suggesting a new therapeutic strategy through hypoxia-related mechanism in AD and PD.

In our study, HPLC were also utilized to pinpoint specific ingredients in YXKFY, which led to the identification of 107 chemical components. The 8 prototype ingredients in the YXKFY-containing serum were found to be quinic acid, lysicamine, riboflavin, cinnamic acid, p-hydroxybenzoic acid, quercetin-7-O-α-L-rhamnoside, tumerone and isoamyl senecioate. Several of these components, including quinic acid, riboflavin, and cinnamic acid, have demonstrated significant anti-inflammatory effects and upregulating antioxidant enzyme activities in neurodegenerative disease. For instance, in in vitro studies, quinic acid has demonstrated neuroprotective and neurotrophic effects on Aβ-induced toxicity, as well as enhancing the activity of neurite outgrowth in PC 12 cells (Hur et al., 2001). It is suggested that quinic acid exerts its neuroprotective effects through the PKA signaling pathway and has successfully restored catalase levels (Rebai et al., 2017). Patients with dementia and AD have shown low levels of riboflavin, and supplementing with riboflavin has been found beneficial in treating cognitive impairment, as it shields cells from oxidative stress by boosting antioxidant enzyme activities and the glutathione redox cycle, and diminishing pro-inflammatory responses in the brain (Zhang et al., 2023). Cinnamic acid, a naturally occurring antioxidant, triggers PPARα activation, promoting lysosomal biogenesis and reducing amyloid plaque pathology in an AD mouse model (Chandra et al., 2019), and protecting dopaminergic neurons in a PD mouse model (Prorok et al., 2019). Therefore, the combination of our discovered results that YXKFY can reduce the ROS levels in hypoxic SH-SY5Y cell injury, suggesting that these components may be effective in YXKFY’s prevention and treatment of AD and PD through antioxidant damage. Moreover, Riboflavin has the best binding affinity with PPIL1, followed by BLVRB, TGM2, FKBPL, and PDGFRB. Additionally, lysicamine and tumerone also exhibit good binding affinity with these proteins. Collectively, the potential therapeutic effects of these chemical monomers from YXKFY on AD and PD through these genes warrant further exploration in our future studies.

## Conclusion

In conclusion, our study leveraged a synergistic approach combining transcriptome and proteome analyses with machine learning to innovatively identify 17 and 3 hypoxia-associated biomarkers for AD and PD, respectively. Furthermore, we elucidated the neuroprotective mechanisms of YXKFY, highlighting its antioxidant properties. This was achieved through the identification of eight bioactive compounds, engagement with five molecular targets, and modulation of several pathways pivotal in neuroprotection.

## Data Availability

The original contributions presented in the study are included in the article/Supplementary Material, further inquiries can be directed to the corresponding authors.

## References

[B1] Aborode A. T. Pustake M. Awuah W. A. Alwerdani M. Shah P. Yarlagadda R. (2022). Targeting oxidative stress mechanisms to treat Alzheimer's and Parkinson's disease: a critical review. Oxid. Med. Cell Longev. 2022, 7934442. 10.1155/2022/7934442 35958022 PMC9357807

[B2] Al-Reza S. M. Yoon J. I. Kim H. J. Kim J. S. Kang S. C. (2010). Anti-inflammatory activity of seed essential oil from Zizyphus jujuba. Food Chem. Toxicol. 48 (2), 639–643. 10.1016/j.fct.2009.11.045 19944733

[B3] Baillieul S. Chacaroun S. Doutreleau S. Detante O. Pépin J. L. Verges S. (2017). Hypoxic conditioning and the central nervous system: a new therapeutic opportunity for brain and spinal cord injuries? Exp. Biol. Med. (Maywood) 242 (11), 1198–1206. 10.1177/1535370217712691 28585890 PMC5478009

[B4] Breijyeh Z. Karaman R. (2020). Comprehensive review on Alzheimer's disease: causes and treatment. Molecules 25 (24), 5789. 10.3390/molecules25245789 33302541 PMC7764106

[B5] Burtscher J. Mallet R. T. Burtscher M. Millet G. P. (2021). Hypoxia and brain aging: neurodegeneration or neuroprotection? Ageing Res. Rev. 68, 101343. 10.1016/j.arr.2021.101343 33862277

[B6] Chai G. Webb A. Lee C. Antaki D. Lee S. Breuss M. W. (2021). Mutations in spliceosomal genes PPIL1 and PRP17 cause neurodegenerative pontocerebellar hypoplasia with microcephaly. Neuron 109 (2), 241–256 e9. 10.1016/j.neuron.2020.10.035 33220177 PMC8800389

[B7] Chandra S. Roy A. Jana M. Pahan K. (2019). Cinnamic acid activates PPARα to stimulate Lysosomal biogenesis and lower Amyloid plaque pathology in an Alzheimer's disease mouse model. Neurobiol. Dis. 124, 379–395. 10.1016/j.nbd.2018.12.007 30578827 PMC6382282

[B8] Chen P. Zhang J. Wang C. Chai Y. H. Wu A. G. Huang N. Y. (2022). The pathogenesis and treatment mechanism of Parkinson's disease from the perspective of traditional Chinese medicine. Phytomedicine 100, 154044. 10.1016/j.phymed.2022.154044 35338993

[B9] Dinkar Gore D. Ahmad F. Tikoo K. Kumar Bansal A. Kumar D. Pal Singh I. (2023). Comparative quantitative analysis of fruit oil from Hippophae rhamnoides (seabuckthorn) by qNMR, FTIR and GC-MS. Chin. Herb. Med. 15 (4), 607–613. 10.1016/j.chmed.2023.05.005 38094022 PMC10715885

[B10] Grabska-Kobyłecka I. Szpakowski P. Król A. Książek-Winiarek D. Kobyłecki A. Głąbiński A. (2023). Polyphenols and their impact on the prevention of neurodegenerative diseases and development. Nutrients 15 (15), 3454. 10.3390/nu15153454 37571391 PMC10420887

[B11] Guo M. Ji X. Liu J. (2022). Hypoxia and alpha-synuclein: inextricable link underlying the pathologic progression of Parkinson's disease. Front. Aging Neurosci. 14, 919343. 10.3389/fnagi.2022.919343 35959288 PMC9360429

[B12] Hayes M. T. (2019). Parkinson's disease and parkinsonism. Am. J. Med. 132 (7), 802–807. 10.1016/j.amjmed.2019.03.001 30890425

[B13] Hur J. Y. Soh Y. Kim B. H. Suk K. Sohn N. W. Kim H. C. (2001). Neuroprotective and neurotrophic effects of quinic acids from Aster scaber in PC12 cells. Biol. Pharm. Bull. 24 (8), 921–924. 10.1248/bpb.24.921 11510486

[B14] Kaeidi A. Taati M. Hajializadeh Z. Jahandari F. Rashidipour M. (2015). Aqueous extract of Zizyphus jujuba fruit attenuates glucose induced neurotoxicity in an *in vitro* model of diabetic neuropathy. Iran. J. Basic Med. Sci. 18 (3), 301–306. 25945244 PMC4414997

[B15] Kim M. J. Jung J. E. Lee S. Cho E. J. Kim H. Y. (2021). Effects of the fermented Zizyphus jujuba in the amyloid β(25-35)-induced Alzheimer's disease mouse model. Nutr. Res. Pract. 15 (2), 173–186. 10.4162/nrp.2021.15.2.173 33841722 PMC8007403

[B16] Korsunsky I. Millard N. Fan J. Slowikowski K. Zhang F. Wei K. (2019). Fast, sensitive and accurate integration of single-cell data with Harmony. Nat. Methods 16 (12), 1289–1296. 10.1038/s41592-019-0619-0 31740819 PMC6884693

[B17] Langfelder P. Horvath S. (2008). WGCNA: an R package for weighted correlation network analysis. BMC Bioinforma. 9, 559. 10.1186/1471-2105-9-559 PMC2631488 19114008

[B18] Lee H. J. Jung Y. H. Choi G. E. Kim J. S. Chae C. W. Lim J. R. (2021). Urolithin A suppresses high glucose-induced neuronal amyloidogenesis by modulating TGM2-dependent ER-mitochondria contacts and calcium homeostasis. Cell Death Differ. 28 (1), 184–202. 10.1038/s41418-020-0593-1 32704090 PMC7852667

[B19] Lee J. Cho E. Kwon H. Jeon J. Jung C. J. Moon M. (2019). The fruit of Crataegus pinnatifida ameliorates memory deficits in β-amyloid protein-induced Alzheimer's disease mouse model. J. Ethnopharmacol. 243, 112107. 10.1016/j.jep.2019.112107 31349027

[B20] Li L. Dong L. Xiao Z. He W. Zhao J. Pan H. (2020). Integrated analysis of the proteome and transcriptome in a MCAO mouse model revealed the molecular landscape during stroke progression. J. Adv. Res. 24, 13–27. 10.1016/j.jare.2020.01.005 32181013 PMC7063112

[B21] Licata L. Lo Surdo P. Iannuccelli M. Palma A. Micarelli E. Perfetto L. (2020). SIGNOR 2.0, the SIGnaling network open resource 2.0: 2019 update. Nucleic Acids Res. 48 (D1), D504–D510. 10.1093/nar/gkz949 31665520 PMC7145695

[B22] Liu Z. P. Wu C. Miao H. Wu H. (2015). RegNetwork: an integrated database of transcriptional and post-transcriptional regulatory networks in human and mouse. Database (Oxford) 2015, bav095. 10.1093/database/bav095 26424082 PMC4589691

[B23] Macdonald R. Barnes K. Hastings C. Mortiboys H. (2018). Mitochondrial abnormalities in Parkinson's disease and Alzheimer's disease: can mitochondria be targeted therapeutically? Biochem. Soc. Trans. 46 (4), 891–909. 10.1042/BST20170501 30026371

[B24] March-Diaz R. Lara-Ureña N. Romero-Molina C. Heras-Garvin A. Ortega-de San Luis C. Alvarez-Vergara M. I. (2021). Hypoxia compromises the mitochondrial metabolism of Alzheimer's disease microglia via HIF1. Nat. Aging 1 (4), 385–399. 10.1038/s43587-021-00054-2 37117599

[B25] Meng F. L. Huang X. L. Qin W. Y. Liu K. B. Wang Y. Li M. (2023). singleCellBase: a high-quality manually curated database of cell markers for single cell annotation across multiple species. Biomark. Res. 11 (1), 83. 10.1186/s40364-023-00523-3 37730627 PMC10510128

[B26] Merelli A. Repetto M. Lazarowski A. Auzmendi J. (2021). Hypoxia, oxidative stress, and inflammation: three faces of neurodegenerative diseases. J. Alzheimers Dis. 82 (1), S109–S126. 10.3233/JAD-201074 33325385

[B27] Miners J. S. Schulz I. Love S. (2018). Differing associations between Aβ accumulation, hypoperfusion, blood-brain barrier dysfunction and loss of PDGFRB pericyte marker in the precuneus and parietal white matter in Alzheimer's disease. J. Cereb. Blood Flow. Metab. 38 (1), 103–115. 10.1177/0271678X17690761 28151041 PMC5757436

[B28] Miterko L. N. Lin T. Zhou J. van der Heijden M. E. Beckinghausen J. White J. J. (2021). Neuromodulation of the cerebellum rescues movement in a mouse model of ataxia. Nat. Commun. 12 (1), 1295. 10.1038/s41467-021-21417-8 33637754 PMC7910465

[B29] Moreira J. Machado M. Dias-Teixeira M. Ferraz R. Delerue-Matos C. Grosso C. (2023). The neuroprotective effect of traditional Chinese medicinal plants-A critical review. Acta Pharm. Sin. B 13 (8), 3208–3237. 10.1016/j.apsb.2023.06.009 37655317 PMC10465969

[B30] Mu H. Q. Liang Z. Q. Xie Q. P. Han W. Yang S. Wang S. B. (2020). Identification of potential crucial genes associated with the pathogenesis and prognosis of prostate cancer. Biomark. Med. 14 (5), 353–369. 10.2217/bmm-2019-0318 32253914

[B31] Nowell J. Blunt E. Edison P. (2023). Incretin and insulin signaling as novel therapeutic targets for Alzheimer's and Parkinson's disease. Mol. Psychiatry 28 (1), 217–229. 10.1038/s41380-022-01792-4 36258018 PMC9812772

[B32] Pan F. Xu X. Zhan Z. Xu Q. (2021). 6-Gingerol protects cardiomyocytes against hypoxia-induced injury by regulating the KCNQ1OT1/miR-340-5p/PI3K/AKT pathway. Panminerva Med. 63 (4), 482–490. 10.23736/S0031-0808.20.03956-7 32720790

[B33] Pei H. Ma L. Cao Y. Wang F. Li Z. Liu N. (2020). Traditional Chinese medicine for Alzheimer's disease and other cognitive impairment: a review. Am. J. Chin. Med. 48 (3), 487–511. 10.1142/S0192415X20500251 32329645

[B34] Prorok T. Jana M. Patel D. Pahan K. (2019). Cinnamic acid protects the nigrostriatum in a mouse model of Parkinson's disease via peroxisome proliferator-activated receptorα. Neurochem. Res. 44 (4), 751–762. 10.1007/s11064-018-02705-0 30612307 PMC6450560

[B35] Rebai O. Belkhir M. Sanchez-Gomez M. V. Matute C. Fattouch S. Amri M. (2017). Differential molecular targets for neuroprotective effect of chlorogenic acid and its related compounds against glutamate induced excitotoxicity and oxidative stress in rat cortical neurons. Neurochem. Res. 42 (12), 3559–3572. 10.1007/s11064-017-2403-9 28948515

[B36] Spooner R. K. Taylor B. K. Moshfegh C. M. Ahmad I. M. Dyball K. N. Emanuel K. (2021). Neuroinflammatory profiles regulated by the redox environment predicted cognitive dysfunction in people living with HIV: a cross-sectional study. EBioMedicine 70, 103487. 10.1016/j.ebiom.2021.103487 34280780 PMC8318860

[B37] Sturrock A. Woller D. Freeman A. Sanders K. Paine R. (2018). Consequences of hypoxia for the pulmonary alveolar epithelial cell innate immune response. J. Immunol. 201 (11), 3411–3420. 10.4049/jimmunol.1701387 30381478 PMC6246786

[B38] Su W. Yang Y. Zhao X. Cheng J. Li Y. Wu S. (2024). Potential efficacy and mechanism of eight mild-natured and bitter-flavored TCMs based on gut microbiota: a review. Chin. Herb. Med. 16 (1), 42–55. 10.1016/j.chmed.2023.08.001 38375054 PMC10874767

[B39] Su Z. Zhang G. Li X. Zhang H. (2023). Inverse correlation between Alzheimer's disease and cancer from the perspective of hypoxia. Neurobiol. Aging 131, 59–73. 10.1016/j.neurobiolaging.2023.07.002 37572528

[B40] Wang L. Liu Z. Liang R. Wang W. Zhu R. Li J. (2022). Comprehensive machine-learning survival framework develops a consensus model in large-scale multicenter cohorts for pancreatic cancer. Elife 11, e80150. 10.7554/eLife.80150 36282174 PMC9596158

[B41] Wang Q. Wang P. Qin Z. Yang X. Pan B. Nie F. (2021). Altered glucose metabolism and cell function in keloid fibroblasts under hypoxia. Redox Biol. 38, 101815. 10.1016/j.redox.2020.101815 33278780 PMC7718484

[B42] Yang H. Cheng J. Zhuang H. Xu H. Wang Y. Zhang T. (2024). Pharmacogenomic profiling of intra-tumor heterogeneity using a large organoid biobank of liver cancer. Cancer Cell 42 (4), 535–551 e8. 10.1016/j.ccell.2024.03.004 38593780

[B43] Yang M. D. Zhou W. J. Chen X. L. Chen J. Ji Q. Li Q. (2021). Therapeutic effect and mechanism of bushen-jianpi-jiedu decoction combined with chemotherapeutic drugs on postoperative colorectal cancer. Front. Pharmacol. 12, 524663. 10.3389/fphar.2021.524663 33828479 PMC8020259

[B44] Yang W. Yin H. Wang Y. Wang Y. Li X. Wang C. (2023). New insights into effects of Kaixin Powder on depression via lipid metabolism related adiponectin signaling pathway. Chin. Herb. Med. 15 (2), 240–250. 10.1016/j.chmed.2022.06.012 37265759 PMC10230638

[B45] Zhang M. Chen H. Zhang W. Liu Y. Ding L. Gong J. (2023). Biomimetic remodeling of microglial riboflavin metabolism ameliorates cognitive impairment by modulating neuroinflammation. Adv. Sci. (Weinh) 10 (12), e2300180. 10.1002/advs.202300180 36799538 PMC10131853

[B46] Zhang S. Ta N. Zhang S. Li S. Zhu X. Kong L. (2024). Unraveling pancreatic ductal adenocarcinoma immune prognostic signature through a naive B cell gene set. Cancer Lett. 594, 216981. 10.1016/j.canlet.2024.216981 38795761

[B47] Zhao H. Liu J. Wang Y. Shao M. Wang L. Tang W. (2023). Polysaccharides from sea buckthorn (Hippophae rhamnoides L.) berries ameliorate cognitive dysfunction in AD mice induced by a combination of d-gal and AlCl(3) by suppressing oxidative stress and inflammation reaction. J. Sci. Food Agric. 103 (12), 6005–6016. 10.1002/jsfa.12673 37132070

[B48] Zhou G. Soufan O. Ewald J. Hancock R. E. W. Basu N. Xia J. (2019b). NetworkAnalyst 3.0: a visual analytics platform for comprehensive gene expression profiling and meta-analysis. Nucleic Acids Res. 47 (W1), W234–W241. 10.1093/nar/gkz240 30931480 PMC6602507

[B49] Zhou Y. Zhou B. Pache L. Chang M. Khodabakhshi A. H. Tanaseichuk O. (2019a). Metascape provides a biologist-oriented resource for the analysis of systems-level datasets. Nat. Commun. 10 (1), 1523. 10.1038/s41467-019-09234-6 30944313 PMC6447622

